# A transcriptional response of *Clostridium beijerinckii* NRRL B-598 to a butanol shock

**DOI:** 10.1186/s13068-019-1584-7

**Published:** 2019-10-13

**Authors:** Karel Sedlar, Jan Kolek, Markus Gruber, Katerina Jureckova, Barbora Branska, Gergely Csaba, Maryna Vasylkivska, Ralf Zimmer, Petra Patakova, Ivo Provaznik

**Affiliations:** 1 Department of Biomedical Engineering, Brno University of Technology, Technicka 12, 616 00 Brno, Czech Republic; 2 Department of Biotechnology, University of Chemistry and Technology Prague, Technicka 5, 166 28 Prague, Czech Republic; 3 Institut für Informatik, Ludwig-Maximilians-Universität München, Amalienstraße 17, 80333 Munich, Germany

**Keywords:** ABE fermentation, Butanol shock, *Clostridium beijerinckii* NRRL B-598, RNA-Seq transcriptome

## Abstract

**Background:**

One of the main obstacles preventing solventogenic clostridia from achieving higher yields in biofuel production is the toxicity of produced solvents. Unfortunately, regulatory mechanisms responsible for the shock response are poorly described on the transcriptomic level. Although the strain *Clostridium beijerinckii* NRRL B-598, a promising butanol producer, has been studied under different conditions in the past, its transcriptional response to a shock caused by butanol in the cultivation medium remains unknown.

**Results:**

In this paper, we present a transcriptional response of the strain during a butanol challenge, caused by the addition of butanol to the cultivation medium at the very end of the acidogenic phase, using RNA-Seq. We resequenced and reassembled the genome sequence of the strain and prepared novel genome and gene ontology annotation to provide the most accurate results. When compared to samples under standard cultivation conditions, samples gathered during butanol shock represented a well-distinguished group. Using reference samples gathered directly before the addition of butanol, we identified genes that were differentially expressed in butanol challenge samples. We determined clusters of 293 down-regulated and 301 up-regulated genes whose expression was affected by the cultivation conditions. Enriched term “RNA binding” among down-regulated genes corresponded to the downturn of translation and the cluster contained a group of small acid-soluble spore proteins. This explained phenotype of the culture that had not sporulated. On the other hand, up-regulated genes were characterized by the term “protein binding” which corresponded to activation of heat-shock proteins that were identified within this cluster.

**Conclusions:**

We provided an overall transcriptional response of the strain *C. beijerinckii* NRRL B-598 to butanol shock, supplemented by auxiliary technologies, including high-pressure liquid chromatography and flow cytometry, to capture the corresponding phenotypic response. We identified genes whose regulation was affected by the addition of butanol to the cultivation medium and inferred related molecular functions that were significantly influenced. Additionally, using high-quality genome assembly and custom-made gene ontology annotation, we demonstrated that this settled terminology, widely used for the analysis of model organisms, could also be applied to non-model organisms and for research in the field of biofuels.

## Background

Solventogenic bacteria from the *Clostridium* genus are used for their ability to produce solvents in acetone–butanol–ethanol (ABE) fermentation [1]. Although it has been more than 100 years, since the first industrial ABE fermentation process was launched, for a long time, bacterial production was replaced by cheaper chemical production from oil [2]. Due to the increasing interest in nature conservation and the fluctuating price of oil, bacterial production of bio-butanol can currently compete with synthetic production [3]. While clostridia represent a large group of organisms with various properties, among the solventogenic representatives three species, *C. acetobutylicum*, *C. beijerinckii*, and *C. pasteurianum*, are primarily of interest in butanol production [4]. This is coupled with the development of molecular tools for manipulation with these species in the last 2 decades, for example ClosTron technology and the modular shuttle plasmids system, transposon-based mutagenesis, counter-selection markers, or CRISPR-Cas-based gene editing [5]. Unfortunately, particular species or even strains can be so different that a tool designed for one strain is not easily applicable to even closely related strains. An example can be found in the strain *C. beijerinckii* NRRL B-598 [6], formerly misidentified as *C. pasteurianum* [7], presented in this study. The strain contains specific restriction–modification (R-M) systems, preventing the use of previously proposed protocols for electrotransformation, conjugation, and sonoporation [8]. Thus, knowledge gathered using the most widely described strains *C. acetobutylicum* ATCC 824 [9], *C. beijerinckii* NCIMB 8052 [10], and *C. pasteurianum* DSM 525 [11] needs to be supplemented by studies of other strains to understand the processes at the molecular level. Even a single-nucleotide variant (SNV) can be responsible for various phenotypic traits [12].

Although various genomes of solventogenic clostridia are studied and compared [13], the genomic sequence itself provides only the theoretical capabilities of an organism and transcriptomic studies are needed to reveal the active parts of a genome. Currently, there are only a few high-quality transcriptomes, which allow full analysis of gene expression and possible post-transcriptional regulation in ABE solventogenic clostridia [4]. For the butanol producing species mentioned above, these mainly include a comprehensive RNome study of *C. acetobutylicum* [14], the transcriptome of *C. beijerinckii* NCIMB 8052 under standard cultivation and with the addition of butyrate into the cultivation medium [15, 16], and our previous transcriptomic studies of *C. beijerinckii* NRRL B-598 under standard cultivation conditions [17, 18]. Therefore, few studies are insufficient to deepen an understanding of butanol production, as solventogenesis is not regulated in the same way, in all solventogenic clostridia and even the same strain can demonstrate different behavior when different cultivation conditions are established [19]. To enhance the knowledge base regarding the behavior of solventogenic clostridia, in this paper, we describe a transcriptional response of *C. beijerinckii* NRRL B-598 to butanol shock caused by the addition of butanol in a concentration of 4.5 g/L to the cultivation medium at the very end of the acidogenic phase. While the transcriptional response to the butanol shock has been mapped for *C. acetobutylicum* [20, 21], it has never been performed for *C. beijerinckii*. Butanol is considered one of the most significant stressors during ABE fermentation [2]; therefore, the butanol challenge experiment was evaluated thoroughly to reveal statistically relevant changes in gene expression. Additionally, we improved the genome assembly by sequencing genomic DNA as our previous study revealed possible misassemblies [18] and reannotated this novel assembly. To summarize the stress response, we utilized gene ontology (GO) enrichment analysis. While this kind of analysis simplifies comparison of responses between various species or strains and can be of great advantage, it is not commonly used for non-model organisms due to lack of comprehensive resources of GO annotation. We scanned various databases and constructed our own high-quality GO annotation. This novel approach can be easily used for other non-model organisms using standard languages for statistical computing. The population heterogeneity was characterized using flow cytometry (FC) coupled with fluorescent staining and, simultaneously, population dynamics and metabolite formation were thoroughly monitored.

## Results

### Cultivation and fermentation kinetics

**Fig. 1:**
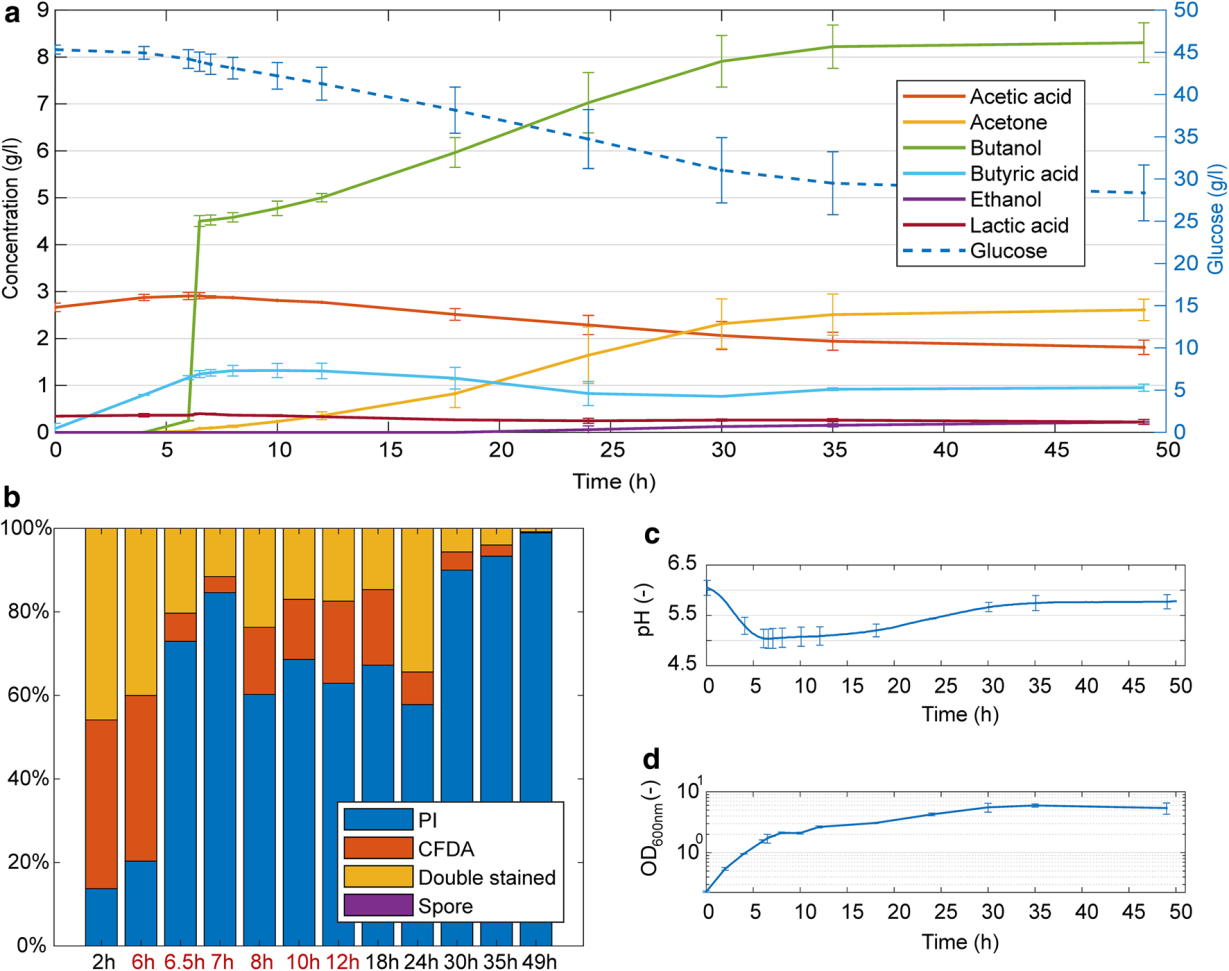
Cultivation and fermentation characteristics of *Clostridium beijerinckii* NRRL B-598 during butanol shock. **a** The concentration of glucose, solvents, and acids during ABE fermentation measured using HPLC. **b** Flow cytometry—the distribution of cells within the population according to their fluorescence pattern for combined staining using PI and CFDA. **c** pH curve for the cultivation. **d** Cell growth measured as optical density at 600 nm. Values represent the mean of the biological replicates and error bars represent the standard deviations. Time-points (*T*_b_0–*T*_b_5) for samples subjected to RNA expression analysis are indicated by red text labels

The goal of the cultivation experiment was to obtain transcriptomic data describing both immediate and later responses towards a non-lethal butanol shock, performed in the phase of transition between the late acidogenic phase and early start of the solventogenesis. Butanol was added directly after sample collection at time 6 h (*T*_b_0). The selected final concentration of added butanol was approximately 0.5% v/v, which was verified previously as unambiguously stressing, but not a lethal concentration for *C. beijerinckii* NRRL B-598 culture [22]. Based on the high-pressure liquid chromatography (HPLC) analyses, there was a small, detectable concentration of butanol produced already before the butanol was added; the exact final concentration of butanol at time 6.5 h (*T*_b_1) was 4.5 g/L (4.42 g/L and 4.58 g/L in the two replicates) (see Fig. 1a). The shock did not stop the butanol production and the next increase in butanol concentration was evident immediately in the sample collected at time 7 h (*T*_b_2). Its production continued until the cultivation was stopped. The final butanol titer was approximately 8.3 g/L (8.0 g/L and 8.6 g/L in the two replicates).

The concentration of all monitored acids (acetic, butyric, and lactic) started to decrease slightly in the culture after the addition of butanol and only the titer of butyric acid started to increase again at time 30 h (see Fig. 1a). Acetone production started around time 6 h and its concentration increased to an approximate time of 35 h. The measured ethanol concentrations were very low at all times as it is typical for this strain also during standard culture conditions [23] (see Fig. 1a and Additional file 1). The butanol shock slowed glucose consumption, compared to standard ABE fermentation. At the end of the cultivation, a relatively high amount of substrate (ca. 30 g/L) remained unused.

After the shock, the cell growth was retarded for approximately the next 4 h, as can be seen in the optical density (OD) analysis (see Fig. 1d). This corresponds well with an increased number of propidium iodide (PI) stained, i.e., non-active, cells identified by FC (see Fig. 1b). After time-point 10 h (*T*_b_4), restored growth of the culture was evident. In the case of the pH course, the culture lacked the traditional rapid increase of pH after the onset of solventogenesis, the so-called metabolic shift (see Fig. 1c and Additional file 1).

The culture produced no spores as determined by a flow cytometry analysis (see Fig. 1b) as well as by light microscopy (Additional file 2). The cells were rod shaped with rather longer chains at the final stages of the experiment. The largest fraction of live cells, carboxyfluorescein diacetate (CFDA) stained, were observed at the beginning of the cultivation prior to the butanol shock at times 2 h and 6 h. Immediately after the addition of butanol, an inhibiting effect was observed. At time 6.5 h as well as 7 h, a number of CFDA stained cells (reflecting those cells with highly active esterases) dropped dramatically and a corresponding increase in cells with damaged cell membrane function, PI stained, was observed. At time 8 h, cell viability was partly restored (39.8% of cells) and the fraction of active cells remained more or less constant up to at least time 24 h. Metabolically active cells were still clearly detectable at time 35 h, but nearly no living cells were found in the last sample (49 h).

### Genome assembly improvement and GO annotation

We used paired-end reads from DNA sequencing for refinement of the previous genome assembly. After adapter and quality trimming, 4 million 150 bp paired-end reads of an overall high quality (average Phred score *Q* ≈ 35) were mapped to the previous CP011966.2 assembly and used for the construction of the augmented assembly, currently available in GenBank under accession number CP011966.3. The novel assembly is 114 bp longer than the previous one (6,186,993 bp vs. 6,186,879 bp). The differences were almost exclusively single-nucleotide changes, except for a single-dinucleotide deletion, and can be divided into three groups: (i) substitutions, (ii) insertions, and (iii) deletions (see Additional file 3). (i) Substitutions affect seven positions, of which four are located in protein-coding regions and the remaining three are in pseudogene regions according to the novel annotation. (ii) Deletions affect seven positions: a single deletion is located in protein-coding region, five in a pseudogene, and the remaining one in an intergenic region. (iii) The largest group is formed of 122 insertions: 86 in protein-coding regions, 31 in intergenic regions, and 5 in pseudogenes. This group is responsible for the majority of changes in the annotation, as in the previous assembly: 75 of these positions were located in pseudogenes, 35 in intergenic regions, 11 in protein-coding regions, and the remaining insertion affected a position where a protein-coding region and a pseudogene overlapped.

The novel assembly was reannotated and the annotation was compared to the previous one (see Table 1). The total number of annotated elements in the augmented assembly is slightly higher, while the number of pseudogenes is reduced. This reduction is caused by a number of insertions mentioned above, resulting in a substantial reduction (100 to 42) of frameshifts detected in pseudogenes. Nevertheless, the changes are not simply caused by the addition of novel loci and the reannotation of pseudogenes as genes (see Additional file 4). In total, 58 loci of the previous assembly were completely discarded from the annotation. The main part, 36 loci, was previously labeled as protein-coding genes, 21 as pseudogenes, and a single locus as non-coding RNA. On the contrary, 68 new loci were introduced in the genome, most of them (44) as pseudogenes and 24 as protein-coding genes. The remaining 96 modifications in the annotation are due to changes of biotypes. While 76 pseudogenes were reannotated as protein-coding genes, 20 protein-coding genes are now labeled as pseudogenes.

**Table 1:** Comparison of genome annotations

	CP011966.2	CP011966.3
Protein-coding genes	5084	5128
RNAs	149	148
Pseudogenes	199	166
Total number of elements	5432	5442

We paid a special attention to the improvement of the GO annotation of the novel assembly. We searched for GO terms assigned to the *C. beijerinckii* NRRL B-598 genome and found 22,013 terms assigned to 3917 distinct genomic elements. Some of these terms were duplicated, since there were four different sources of annotation: UniProt [24], InterPro [25], Gene Ontology Consortium (GOC) [26], and RNAcentral [27]. After the removal of duplications, 16,271 uniquely assigned terms remained in the annotation. The remaining genomic elements, without any assigned GO term, were subjected to sequence-based annotation in InterPro and GO databases. To find relevant homologies, protein BLAST [28] searches against the whole bacterial domain were used. After filtering out duplications and obsolete terms, 1702 distinct GO terms were assigned to 4455 genomic elements in 18,020 unique assignments. The resulting annotation was summarized in a map file (see Additional file 5) that can be used for GO enrichment analysis in the R/Bioconductor package topGO [29]. We also added a brief overview of the GO annotation by assigning levels (their longest distance from the root) to assigned terms (see Additional file 6). The most common term is GO:0016021 “integral component of membrane”, from the cellular component (CC) category, assigned to 1251 genes. The most abundant terms from the biological process (BP) and molecular function (MF) categories are GO:0055114 “oxidation–reduction process” with 430 genes and GO:0016740 “transferase activity” with 610 genes, respectively. Nevertheless, these values are extreme and a median value of the times of a GO term assignment is two.

### RNA-Seq transcriptome

Our RNA-Seq data set of *C. beijerinckii* NRRL B-598 response to a butanol shock covers six time-points (*T*_b_0–*T*_b_5) by two independent biological replicates, labeled as F and G (as we continue to label our RNA-Seq samples of the strain in alphabetical order, A–E were assigned to standard ABE fermentation in our previous studies [17, 18]). The whole data set contains almost 450 million 75 bp single-end reads. Despite the rRNA depletion performed prior to the library construction, reads corresponding to rRNA were detected and removed prior to the mapping in silico. The amount of remaining non-rRNA reads ranged from 1.4 to 5.3 million per sample (see Additional file 7). Although the quality assessment after the first preprocessing steps (demultiplexing, quality trimming, and adapter trimming) confirmed an overall high-quality of sequences (average Phred score *Q* ≈ 35), in some samples, almost 20% of reads could not have been mapped unambiguously (see Additional file 7). Reads mapping to the genome more than ten times were discarded and counted as unmapped. To cover the expression of duplicated genes, the reads mapping to the genome up to ten times were included in the gene expression analysis (see Table 2). However, the contribution of such reads was down-weighted in the expression analysis, depending on the number of times they mapped to the genome, so the sum of the number of counted reads remained the same. Similarly, reads mapping to more than one genomic object were also down-weighted. In the current assembly, there are 311 overlapping loci. The majority of them are formed by 294 pairs of overlapping protein-coding genes, the additional 16 genes overlap with pseudogenes, and the remaining single case corresponds to two overlapping pseudogenes. In total, 33 protein-coding genes and four pseudogenes demonstrated no transcripts (RPKM < 1) at any of the six sampling points.

**Table 2:** Transcriptional activity of genes and pseudogenes

Sample	*T*_b_0 (6 h)	*T*_b_1 (6.5 h)	*T*_b_2 (7 h)	*T*_b_3 (8 h)	*T*_b_4 (10 h)	*T*_b_5 (12 h)	Total
No. of genes with RPKM > 1^a^	4942 (4891)	4943 (4888)	4967 (4907)	4972 (4918)	5003 (4951)	5003 (4968)	5095 (5054)
No. of pseudogenes with RPKM > 1^a^	112 (141)	147 (142)	146 (143)	147 (144)	152 (148)	147 (142)	162 (160)
Max. expression (RPKM)	4.5 × 10^4^	8.2 × 10^4^	6.3 × 10^4^	7.8 × 10^4^	7.7 × 10^4^	8.0 × 10^4^	8.2 × 10^4^

^a^Values in brackets apply to uniquely mapped reads only

**Fig. 2:**
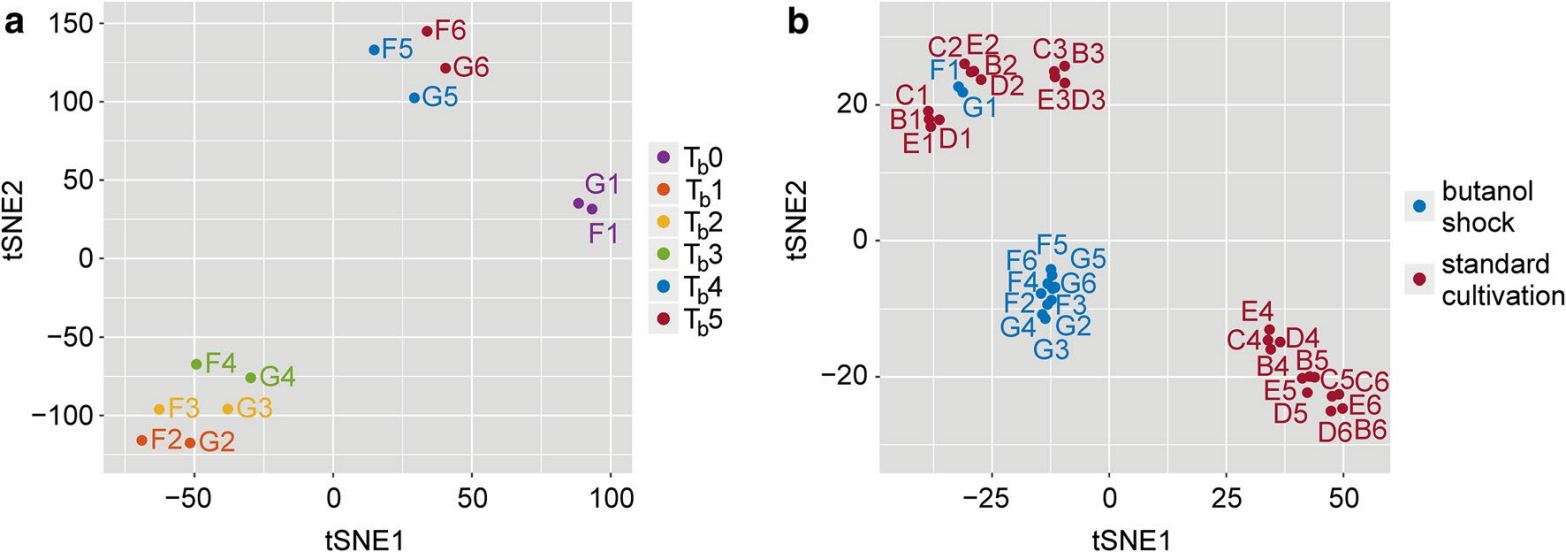
Overall comparison of RNA-Seq samples. 2D representation of the normalized expression data after dimensionality reduction by t-SNE. **a** Comparison of the samples collected at the six time-points (*T*_b_0–*T*_b_5) coded by different colors. Each point represents a sample with a text label indicating the biological replicate (F, G) and the time-point from which it originated (*T*_b_0–*T*_b_5). **b** Comparison of the samples collected during butanol shock cultivation (red) and the samples from our previous studies [17, 18] during standard cultivation (blue). Again, points represent samples with a text labels indicating biological replicates (B, C, D, and E for standard cultivation and F and G for butanol shock). Samples F1 and G1 collected before butanol addition at time-point *T*_b_0 = 6 h correspond to samples B2, C2, D2, and E2 collected at *T*2 = 6 h during standard cultivation

Reproducibility of the experiment was supported by the utilization of two biological replicates and by the comparison of replicates to the previously gathered data sets. An overview of the data set produced by the t-Distributed Stochastic Neighbor Embedding (t-SNE) [30] dimensionality reduction method applied to the normalized expression data suggested a partitioning of the samples into three separate clusters (see Fig. 2a). The first was formed by samples obtained directly before butanol addition to the cultivation medium. Samples from the following three time-points formed the second cluster and samples from the remaining two time-points formed the third cluster. Differences between samples before and after butanol addition are particularly visible in comparison to previously gathered samples during standard cultivation [17, 18] (see Fig. 2b). While samples before butanol addition cluster to the corresponding samples from standard cultivation, samples after butanol addition form a separate cluster. To perform the comparison, we mapped samples from the previous studies to the novel genome assembly CP011966.3.

### Differential expression

**Fig. 3:**
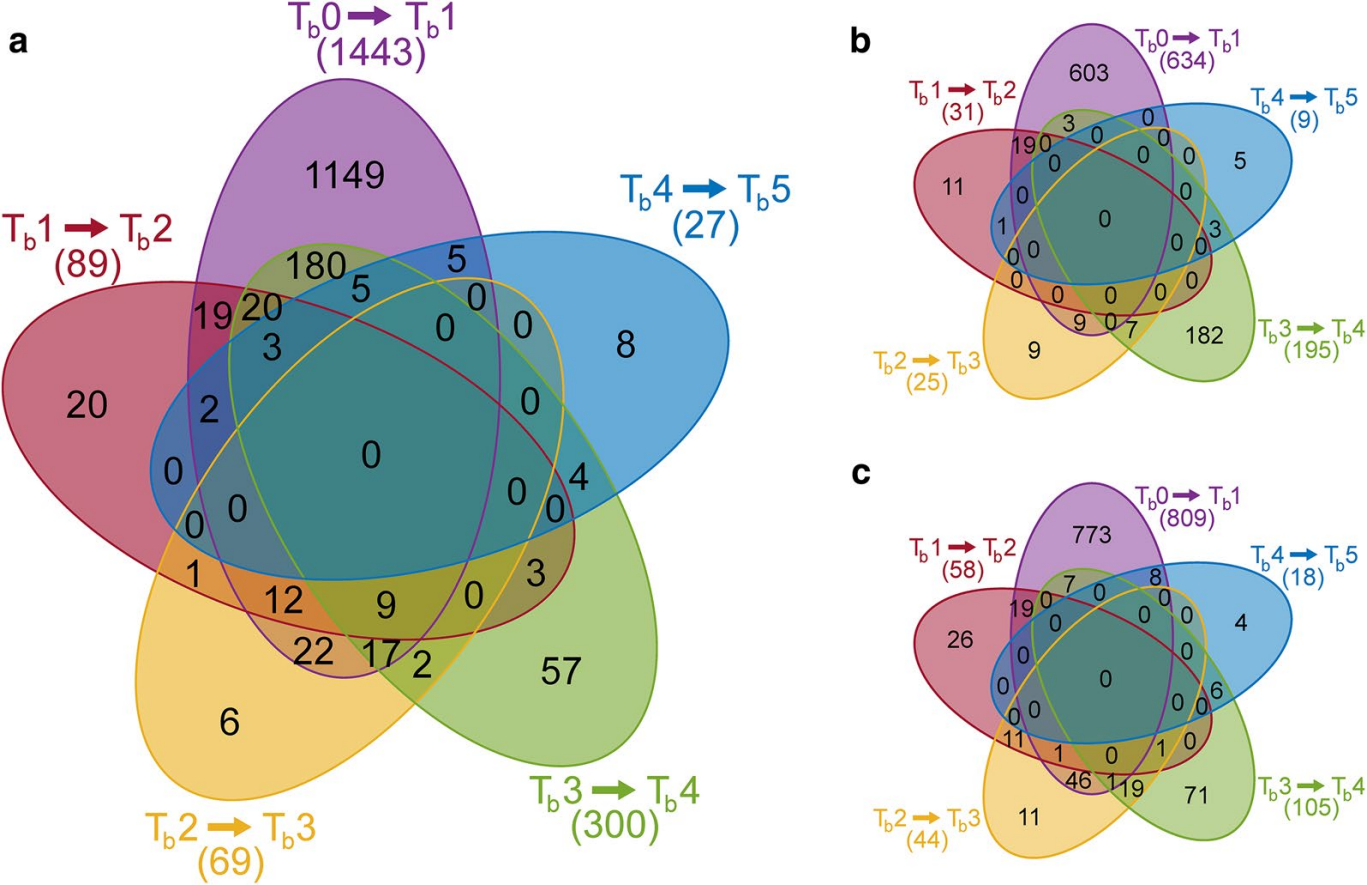
Differential expression analysis of adjacent time-points. Venn diagrams showing the number of **a** all-regulated, **b** up-regulated, and **c** down-regulated genomic elements between adjacent time-points

To further analyze particular samples, we performed differential expression analysis of adjacent time-points and showed the results as respective Venn diagrams (see Fig. 3 and Additional file 8). In accordance with the previous dimensionality reduction, the main regulation was detected directly after butanol addition (between *T*_b_0 and *T*_b_1), when 1443 loci were regulated (adjusted *p* value < 0.05, Benjamini–Hochberg correction) and the second highest regulation between *T*_b_3 and *T*_b_4, when 300 loci were differentially expressed. In total, 1499 protein-coding genes were regulated at least once between adjacent time-points, 303 of these more than once. The remaining 3629 protein-coding genes had no statistically significant regulations among adjacent time-points. Only 14 out of 166 pseudogenes were regulated, 13 were regulated once, and a single pseudogene was regulated twice. Only a single non-coding RNA gene X276_26885 was regulated once, directly after butanol addition. The complete results of the differential expression analysis among adjacent time-points, including log2FoldChanges and adjusted *p* values, are available in Additional file 9.

**Fig. 4:**
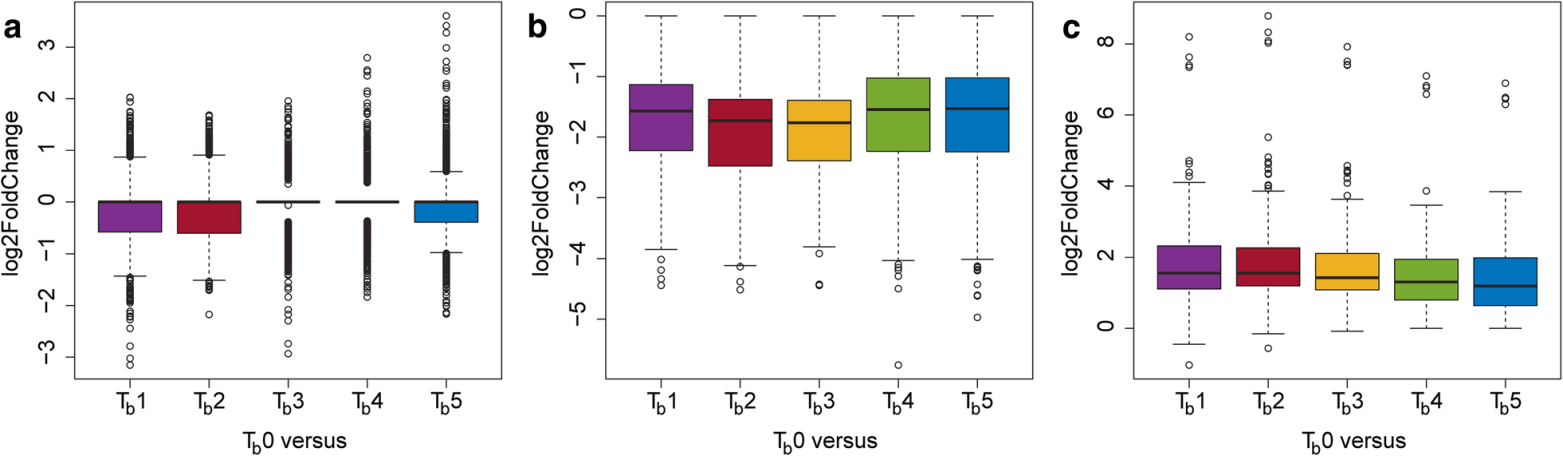
Changes in expression of regulated genes in comparison to the reference time-point. Boxplots showing log2FoldChanges in expression of genes that, as compared to the reference time-point, are **a** non-regulated (cluster 1), **b** down-regulated (cluster 2), and **c** up-regulated (cluster 3)

We explored differentially expressed genes at particular time-points against the reference time-point *T*_b_0, prior to the butanol addition, to find gene expression changes elicited by butanol addition. There were 2037 genomic loci with at least one statistically significant differential expression (adjusted *p* value < 0.05, Benjamini–Hochberg correction). Based on their log2FoldChanges in all five comparisons, genes were distributed into three clusters. Although all selected loci had at least one significant change in expression, loci within the first cluster of 1443 elements demonstrated zero log2FoldChanges on average. Genes within the second (293 elements) and the third cluster (301 elements) are significantly down-regulated and up-regulated, respectively (see Fig. 4). While the first cluster also captures noise and contains loci of various biotypes, including four rRNA genes, the second cluster of down-regulated elements is formed exclusively by protein-coding genes. The third cluster of up-regulated elements is formed mainly by protein-coding genes, but it also contains nine pseudogenes, a single non-coding RNA gene, and a tRNA gene.

### Gene ontology enrichment

To explore and describe the functional response to the butanol shock, we performed MF GO enrichment analysis in all three clusters of genes using all 2037 regulated genomic loci as the gene universe. MF GO terms significantly enriched (*p* value < 0.05, Fisher’s exact test) in cluster 1 were especially terms related to “iron ion binding”, “methyltransferase”, “nuclease activity”, “helicase activity”, and others (see Table 3). Among the genes annotated with the term “iron ion binding” are genes for ferredoxin, acyl-CoA-dehydrogenase, genes involved in Fe–S proteins biosynthesis, pyruvate:ferredoxin (flavodoxin) oxidoreductase, and many more genes which are indispensable or house-keeping (see Additional file 10).

**Table 3:** GO enrichment results in cluster 1

GO.ID	Term	Annotated	Significant	Expected	classicFisher
GO:0004518	Nuclease activity	19	18	13.42	0.012
GO:0004386	Helicase activity	18	17	12.71	0.016
GO:0016741	Transferase activity, transferring one-carbon groups	55	46	38.85	0.019
GO:0043169	Cation binding	211	162	149.03	0.019
GO:0046872	Metal ion binding	207	159	146.21	0.020
GO:0010181	FMN binding	17	16	12.01	0.021
GO:0004519	Endonuclease activity	11	11	7.77	0.021
GO:0005506	Iron ion binding	22	20	15.54	0.023
GO:0008168	Methyltransferase activity	45	38	31.78	0.024
GO:0046914	Transition metal ion binding	74	59	52.27	0.048

**Fig. 5:**
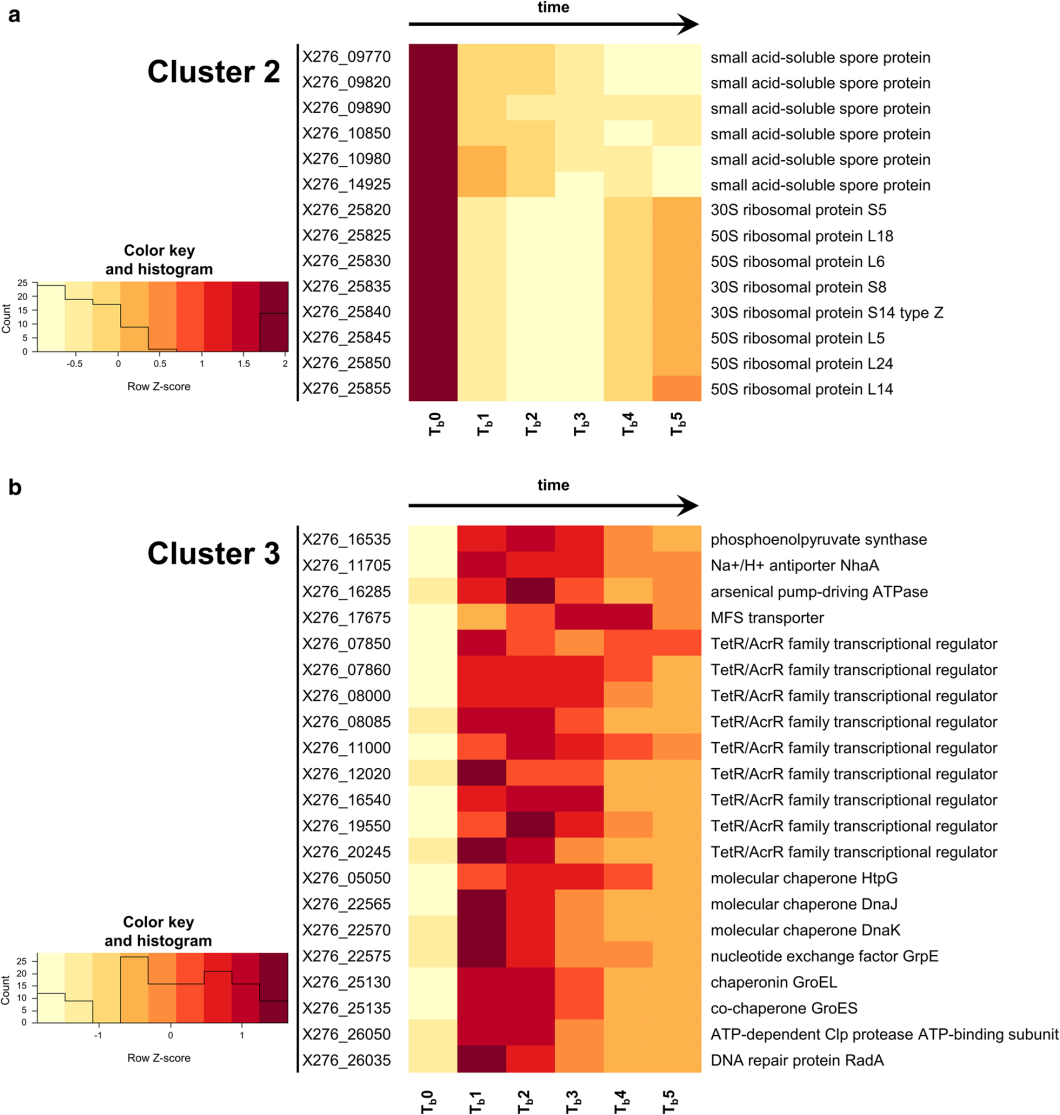
Expression profiles of selected genes. Heatmap showing transcriptional profiles of selected genes within **a** cluster 2 and **b** cluster 3 using *Z* scores computed from the distribution of expression values of each gene

In cluster 2 (down-regulated), we can recognize as main recurring terms “dsDNA binding”, “RNA/rRNA binding”, and several terms which are connected to transports like “ATPase activity”, “amine transmembrane transporter activity”, “organic acid transmembrane transporter”, or “anion/organic anion transmembrane transporter” (see Table 4). Under term “ATPase activity”, we can distinguish many ABC transporters with various functions. Reflecting growth attenuation, down-regulation of distinctive group of genes involved in proteosynthesis like ribosome components (see Fig. 5 and Additional file 11) can be found in terms referring to “structural constituent of ribosome”, “structural molecule activity”, and “RNA/rRNA binding”. Aborted preparation for sporulation is connected with down-regulation of group of genes coding small acid-soluble spore proteins (see Fig. 5 and Additional file 11), which can be found associated with term “dsDNA binding.”

**Table 4:** GO enrichment results in cluster 2

GO.ID	Term	Annotated	Significant	Expected	classicFisher
GO:0003735	Structural constituent of ribosome	54	22	7.38	4.70E−07
GO:0005198	Structural molecule activity	56	22	7.65	9.90E−07
GO:0019843	rRNA binding	37	17	5.06	1.40E−06
GO:1901682	Sulfur compound transmembrane transporter activity	7	6	0.96	3.80E−05
GO:0015116	Sulfate transmembrane transporter activity	5	5	0.68	4.60E−05
GO:0015419	ATPase-coupled sulfate transmembrane transporter activity	5	5	0.68	4.60E−05
GO:0031177	Phosphopantetheine binding	6	5	0.82	0.00024
GO:0072341	Modified amino acid binding	6	5	0.82	0.00024
GO:0008509	Anion transmembrane transporter activity	23	10	3.14	0.00041
GO:0003690	Double-stranded DNA binding	10	6	1.37	0.00079
GO:0043225	ATPase-coupled inorganic anion transmembrane transporter activity	8	5	1.09	0.00179
GO:0003723	RNA binding	78	20	10.66	0.00269
GO:0022857	Transmembrane transporter activity	141	31	19.27	0.00298
GO:0005215	Transporter activity	148	32	20.22	0.00338
GO:0005275	Amine transmembrane transporter activity	6	4	0.82	0.00406
GO:0015424	Amino acid-transporting ATPase activity	6	4	0.82	0.00406
GO:0031263	Amine-transporting ATPase activity	6	4	0.82	0.00406
GO:0033283	Organic acid-transporting ATPase activity	6	4	0.82	0.00406
GO:0033284	Carboxylic acid-transporting ATPase activity	6	4	0.82	0.00406
GO:0015318	Inorganic molecular entity transmembrane transporter activity	55	15	7.52	0.00493
GO:0015103	Inorganic molecular entity transmembrane transporter activity	10	5	1.37	0.00639
GO:0015171	Amino acid transmembrane transporter activity	10	5	1.37	0.00639
GO:0016765	Transferase activity, transferring alkyl or aryl (other than methyl) groups	10	5	1.37	0.00639
GO:0033218	Amide binding	10	5	1.37	0.00639
GO:0004794	l-Threonine ammonia-lyase activity	4	3	0.55	0.00905
GO:0015075	Ion transmembrane transporter activity	60	15	8.2	0.0117
GO:0042626	ATPase activity, coupled to transmembrane movement of substances	39	11	5.33	0.01196
GO:0043492	ATPase activity, coupled to movement of substances	39	11	5.33	0.01196
GO:0005342	Organic acid transmembrane transporter activity	12	5	1.64	0.01594
GO:0046943	Carboxylic acid transmembrane transporter activity	12	5	1.64	0.01594
GO:0004124	Cysteine synthase activity	2	2	0.27	0.01859
GO:0004421	Hydroxymethylglutaryl-CoA synthase activity	2	2	0.27	0.01859
GO:0004779	Sulfate adenylyltransferase activity	2	2	0.27	0.01859
GO:0004781	Sulfate adenylyltransferase (ATP) activity	2	2	0.27	0.01859
GO:0015087	Cobalt ion transmembrane transporter activity	2	2	0.27	0.01859
GO:0016887	ATPase activity	92	20	12.57	0.01898
GO:0008514	Organic anion transmembrane transporter activity	13	5	1.78	0.02308
GO:0015399	Primary active transmembrane transporter activity	44	11	6.01	0.0294
GO:0015405	P–P-bond-hydrolysis-driven transmembrane transporter activity	44	11	6.01	0.0294
GO:0019842	Vitamin binding	39	10	5.33	0.03149
GO:0008982	Protein-N(PI)-phosphohistidine-sugar phosphotransferase activity	14	5	1.91	0.03201
GO:0015144	Carbohydrate transmembrane transporter activity	14	5	1.91	0.03201
GO:0016841	Ammonia-lyase activity	6	3	0.82	0.03666
GO:0022804	Active transmembrane transporter activity	76	16	10.38	0.04519

Up-regulated genes in cluster 3 are significantly enriched in terms like “transcriptional regulation”, “protein binding”, or “ATP binding” (see Table 5). GO term “secondary active transport” is also significantly enriched. The third cluster contains genes coding molecular chaperones like DnaKJ, GroESL, HptG, and several other heat-shock proteins (HSPs), which can be found associated with the term “protein/ATP binding” (see Fig. 5 and Additional file 12). A large group of genes coding putative TetR/AcrR regulation factors are also part of cluster 3 and term “DNA binding” (see Fig. 5 and Additional file 12); ctsR, hrcA, or putative sigma factors can also be found in the same group.

**Table 5:** GO enrichment results in cluster 3

GO.ID	Term	Annotated	Significant	Expected	classicFisher
GO:0003677	DNA binding	221	57	34.7	1.70E−05
GO:0004803	Transposase activity	8	6	1.26	3.00E−04
GO:0005515	Protein binding	33	13	5.18	0.00077
GO:0008519	Ammonium transmembrane transporter activity	5	4	0.79	0.00261
GO:0051082	Unfolded protein binding	5	4	0.79	0.00261
GO:0050567	Glutaminyl-tRNA synthase (glutamine-hydrolyzing) activity	3	3	0.47	0.00383
GO:0003700	DNA-binding transcription factor activity	76	20	11.93	0.01011
GO:0140110	Transcription regulator activity	77	20	12.09	0.01176
GO:0005315	Inorganic phosphate transmembrane transporter activity	4	3	0.63	0.01354
GO:0030554	Adenyl nucleotide binding	233	48	36.59	0.01813
GO:0000150	Recombinase activity	2	2	0.31	0.02457
GO:0004139	Deoxyribose-phosphate aldolase activity	2	2	0.31	0.02457
GO:0008880	Glucuronate isomerase activity	2	2	0.31	0.02457
GO:0005488	Binding	802	140	125.94	0.02519
GO:0005524	ATP binding	232	47	36.43	0.02616
GO:0032559	Adenyl ribonucleotide binding	232	47	36.43	0.02616
GO:0097159	Organic cyclic compound binding	625	112	98.15	0.02678
GO:1901363	Heterocyclic compound binding	625	112	98.15	0.02678
GO:0046983	Protein dimerization activity	5	3	0.79	0.02993
GO:0003676	Nucleic acid binding	316	61	49.62	0.03063
GO:0016879	Ligase activity, forming carbon–nitrogen bonds	30	9	4.71	0.03468
GO:0140097	Catalytic activity, acting on DNA	36	10	5.65	0.04418
GO:0015291	Secondary active transmembrane transporter activity	22	7	3.45	0.0446

## Discussion

Although the previous version of the genome CP011966.2 was reconstructed using a combination of next generation sequencing and third-generation sequencing, the assembly suffered from the inability of Roche 454 pyrosequencing to adjust low-quality PacBio RSII sequencing, especially in homopolymeric regions of the genome [31]. This was apparent from our previous transcriptomic study of the strain, where Illumina sequencing revealed possible indels in coding regions [18]. Therefore, we decided to employ additional DNA sequencing, since even an SNV can be responsible for substantial phenotypic differences in solventogenic clostridia [12, 32]. A number of insertions and deletions introduced in the novel version of the genome CP011966.3 (see Additional file 3) confirmed errors in the homopolymeric regions and led to the substantial reduction of frameshifts in detected open reading frames and to the overall reduction in a number of genomic elements annotated as pseudogenes. Moreover, all 12 insertions and three nonsynonymous substitutions in protein-coding sequences resulted in proteins more similar to other proteins produced by bacteria from the *Clostridium* genus. The annotation of the augmented genome sequence introduced several changes (see Additional file 4). A number of elements coding hypothetical proteins were reduced as 48 of these elements were discarded from the genome and only 26 were newly introduced. An additional 14 hypothetical proteins were identified by changes in pseudogenes. Twenty-two of the twenty-three pseudogenes that were selected as putative active genes in our previous study by Sedlar et al. [18] were automatically reannotated as protein-coding genes due to the changes in the augmented assembly. Thus, the current version of the genome confirmed our previous findings.

Even though BLAST-based GO annotation tends to capture all true assignments, its overall precision is hampered by a number of false positive assignments [33]. We reduced possible misannotations by merging BLAST-based annotation with InterPro annotation, which has higher precision, yet lower recall, in Blast2GO suite [34]. Our manually curated annotation shows a distribution of GO term levels very similar to the annotation reconstructed from database searches only (see Additional file 6) and the median value of the times of a GO term assignment is the same. Although purely computationally inferred GO annotations are sufficient for many analyses [35], we consider our curation steps to be a quality improvement. While dimensionality reduction of butanol shock data suggested division of time-points into three clusters (see Fig. 2a), differences between clusters formed by *T*_b_1–*T*_b_3 and *T*_b_4–*T*_b_5 time-points are not so evident when the whole data set is compared to the RNA-Seq data set from a standard cultivation (see Fig. 2b). The visible difference between samples from the first time-point *T*_b_0 to those at the remaining time-points was supported by differential expression analysis, when the number of regulated genes was the highest (see Fig. 3a). The second highest number of differentially expressed genes was recorded between *T*_b_3 and *T*_b_4 time-points, and confirmed the difference between *T*_b_1–*T*_b_3 and *T*_b_4–*T*_b_5 clusters. While the difference between *T*_b_0 and *T*_b_1–*T*_b_3 can be accredited to a defense reaction to butanol shock, an increased number of regulated genes between *T*_b_3 and *T*_b_4 are connected to the restored growth of population. Even though it was reported that viability of *C. beijerinckii* NRRL B-598 was not altered when a butanol challenge of approximately 5 g/L was added prior to inoculation [36], the addition of butanol at a late acidogenic stage induced a loss of vital function in a significantly high number of cells. This, together with abandoned sporulation, is probably the reasons that *T*_b_4 and *T*_b_5 samples did not cluster with the respective stage from standard cultivation, even though no negative regulation or any visible interference between butanol addition and production was observed. This correlates with results obtained for *C. acetobutylicum* [20, 21], where butanol addition up-regulated its synthesis.

The final butanol titer at the end of cultivation was approximately 8.3 g/L including added butanol, which means that the final concentration of produced butanol was roughly 4 g/L. This indicates that, in butanol challenge cultivation, butanol probably reached the maximally tolerated titer for metabolic activity of the cells, such that further butanol production has been inhibited. A similar maximal concentration was also reached using *C. beijerinckii* NRRL B-598 during the same butanol shock, but with an initial glucose concentration 20 g/L [22].

To summarize the response to a butanol shock, we used our novel GO annotation (Additional file 5) to perform a GO enrichment analysis. Pairwise comparison of the samples measured before butanol addition with samples after butanol addition allowed us to focus on the subset of genes that were differentially expressed because of butanol addition. While the total number of differentially expressed genes was relatively high (2037), log2FoldChange-based clustering revealed further division of these genes into three clusters. The first and the largest cluster of 1443 genes demonstrated high variance of values and a lot of outliers, but almost zero median value. Therefore, we consider these genes as non-regulated due to the butanol shock. Statistically significant differential expressions in this cluster are like due to noise, biological as well as technical. First, the cell cycle within the culture is unsynchronized, and thus, regulations of genes that were not caused by the butanol shock can be captured. Second, there is technical noise remaining in the data. Although the data were carefully filtered, contaminations always remain. This is apparent, for example, from four regulated rRNA genes within the first cluster caused by remaining rRNA reads. While the number of reads mapping to rRNA loci is very low, similarly low changes in their abundance between different samples can be incorrectly identified as differential expression. The truly down- and up-regulated genes due to the butanol shock can be found in cluster 2 and cluster 3, respectively. Both clusters contain around 300 genes (293 and 301, respectively), which are only small fractions of the total number of genes in the genome of *C. beijerinckii* NRRL B-598 suitable for proper GO enrichment analysis during the butanol shock.

Although cluster 1 contained genes that were likely not regulated by the butanol shock, we decided to perform a GO enrichment analysis to summarize these genes. The cluster was formed by a mixture of genes with various functions, which resulted in only ten significantly enriched GO terms at the significance level *α* = 0.05. Moreover, no *p* value of Fisher’s exact test was lower than 0.01. Further inspection of genes associated with enriched GO terms revealed that some of these genes are probably indispensable, house-keeping (see Additional file 10), or coding enzymes necessary for DNA maintenance (e.g., DNA polymerase, primase, helicase, topoisomerase, or methyltransferase).

GO enrichment analysis in clusters of down-regulated (cluster 2) and up-regulated (cluster 3) genes revealed similar physiological response as described by Alsaker et al. [21], where global response was expressed as representation of differentially expressed genes in different clusters of orthologous genes (COG) categories. Among others, GO terms like “structural constituent of ribosome” (GO:0003735), “structural molecule activity” (GO:0005198), and “RNA/rRNA binding” (GO:0003723/GO:0019843) were enriched in cluster 2, which is in accordance with the significant down-regulation in COG category J (translation) for *C. acetobutylicum* [21]. Enrichment of these terms is caused by a group of genes that are assigned a couple of GO terms, even all of these four GO terms. These terms are close neighbors in the GO graph, which hints at the possibility of further slimming the GO annotation for solventogenic clostridia in the future. The highest percentage of up-regulated genes after butanol addition to *C. acetobutylicum* culture was found in COG category O (post-translational modification, protein turnover, and chaperones) [21]. Similarly, up-regulated HSPs in our study can be found associated with the GO term “protein/ATP binding” (GO:0005515/GO:0005524) in the GO enrichment analysis of cluster 3. HSPs are able to help with protein folding to native conformation, dsDNA stabilization, or can induce next changes in expression in the role of stress transcription factors [37]. Expression of HSPs during butanol production or butanol shock has been previously described in many works [2, 38–40] and several HSPs are the most probably involved in butanol stress reaction *C. beijerinckii* NRRL B-598, as well [17]. During standard cultivation, it was shown that production of class I HSPs, including DnaKJ and GroESL, were particularly regulated by pH stress and acid production, while genes coding alternative sigma-factor SigI, related theoretically to class II HSPs expression, were regulated in accordance with highest butanol titer. Similarly, genes for class III HSPs and uncategorized HSP HptG were also highly expressed when butanol started to be produced in higher concentrations [17]. Strong up-regulation of *dnaK*, *dnaJ*, *groES*, *groEL*, *grpE*, *radA*, or *hptG* was also evident after butanol addition during butanol challenge cultivation (see Fig. 5). This fully supports the premise and already published results obtained for *C. acetobutylicum* [20, 21] that HSPs play a fundamental role in overcoming butanol stress. Although some GO terms may appear generic, their connection to butanol tolerance is meaningful. For example, term “DNA-binding transcription” factor activity (GO:0003700) was also found to be enriched during *n*-butanol challenge in *Escherichia coli* [41].

It is evident from FC analysis and microscopy that culture did not produce any matured spores, prespores, or even thick, so-called “clostridial” cells accumulating granulose during cultivations with butanol addition (see Fig. 1b and Additional file 2). This is, as expected, in contrast to standard cultivation experiments under the same cultivation conditions (see Additional file 1) [17] and also does not correlate with the response of *C. acetobutylicum* to butanol shock [20, 21], where sporulation remained unaffected. Moreover, sporulation suppression and, at the same time, intact solventogenesis can be considered another evidence for independent regulation of sporulation and solventogenesis in *C. beijerinckii* NRRL B-598, which fully correlates with already published results [17, 19, 36]. The fact that sporulation was not induced could have been caused by relatively small final density of cells in comparison with standard cultivation (see Additional file 1). An Agr-based quorum sensing system can be responsible for the initiation of granulose formation and subsequent sporulation in solventogenic clostridia, as postulated previously [42]. The differences in butanol elicited stress response in *C. beijerinckii* NRRL B-598, and *C. acetobutylicum* ATCC 824 might result in different organization of Agr quorum sensing genes in both genomes and no found homologies in the respective genes in both strains [17, 43]. Thus, quorum sensing could be a reason why sporulation was not started and, therefore, several genes related to spore formation were found in cluster 2. Apparent down-regulation was detected for small, acid-soluble proteins (SASPs), small proteins coating DNA in matured spores with putative peroxidase activity, which play a fundamental role in DNA protection [44, 45]. Observed expression of SASPs is in contrast with standard expression of SASPs in *C. perfringens* where SASPs are expressed after the start of sporulation [46] and are expressed under regulation of *sigG* and *sigF* in *C. acetobutylicum* [47]. On the other hand, Wetzel et al. [47] assert that SASPs can bind DNA in vitro which implies that SASPs could potentially protect DNA against nucleases, not only in matured spores.

## Conclusions

Mechanisms preventing solventogenic clostridia from producing a higher titer of biofuels are widely studied yet remain unclarified. There are several reasons for this. First, solventogenic clostridia are non-model organisms whose genome sequences started to be explored only recently. Although genomes of more and more strains are being sequenced and assembled, only a few of them are robustly assembled using various sequencing techniques to fix assembly errors caused by specific biases or errors. Since even single-nucleotide changes in genomic sequences are responsible for various phenotypic traits, comparison of different strains may be difficult. Second, there is a lack of further exploration of different strains under various cultivation conditions. Moreover, a unified annotation summarizing behavior of various strains or a selected strain under different conditions is missing. Here, we overcame these obstacles by resequencing the genome of *C. beijerinckii* NRRL B-598 to produce the high-quality assembly with unified GO annotation and by exploring the transcriptional processes during butanol challenge cultivation using RNA-Seq and auxiliary HPLC and FC techniques.

The main change in transcriptional regulation was captured directly after butanol addition. When compared to the samples from a standard cultivation, samples from a butanol challenge forms a distinguished group. Still, they can be further divided into two groups. The first group is formed by samples obtained within 2 h after butanol addition and can be assigned to a defense reaction to the butanol shock. The second group captures samples where growth of population was restored; still expression of genes is different from the standard cultivation samples. To summarize the transcriptional response connected to the butanol shock, we selected only genes that are differentially expressed in a majority of pairwise comparisons of samples gathered during butanol challenge to samples gathered before butanol addition. We utilized our custom-made GO annotation to characterize the clusters of up- and down-regulated genes. This allowed us to describe the response to the butanol shock in detail using a well-defined terminology. Moreover, this analysis has been compared to a somewhat coarser analysis of the response of *C. acetobutylicum* to a butanol shock using clusters of orthologous genes. The butanol response in both species resulted in up-regulation of heat-shock protein genes and did not intervene with solventogenesis. On the other hand, there was a significant difference in sporulation. While sporulation and also granulose formation were suppressed in *C. beijerinckii* NRRL B-598, these life cycle events remained unaffected in *C. acetobutylicum* which may serve as further indirect evidence for uncoupling sporulation and solventogenesis regulation in *C. beijerinckii* NRRL B-598. We believe that the proposed novel high-quality assembly and annotation will be very useful for the future exploration of the strain and will inspire others to start using this well-defined terminology when describing transcriptional responses of solventogenic clostridia.

## Methods

### Bacterial culture and fermentation experiment

Culture of the strain *C. beijerinckii* NRRL B-598 was obtained from NRRL (ARS) collection of microorganisms and was maintained as a spore suspension in 4 °C in distilled water. For all manipulation, TYA broth [19] containing 20 g/L or 50 g/L of glucose was used. The bacterial strain was cultivated in parallel Multifors 1 L bioreactors (INFORS HT, Bottmingen, Switzerland). Preparation process of the culture inoculum and initial cultivation parameters were chosen the same as in Patakova et al. [17]. At the beginning of cultivation, pH of the culture was adjusted to 6.3 by NaOH solution addition and pH was monitored, but not controlled during the following cultivation.

Directly after collection of samples at time 6 h of cultivation, butanol shock was performed by addition of pure, HPLC-grade butanol (Sigma-Aldrich, Praha, Czechia) to final concentration approximately 0.5% v/v. Control sampling prior to and after addition were conducted for specification of precise added butanol concentration. Butanol was added to the bioreactor under strictly sterile and anaerobic conditions.

### Culture growth and HPLC analysis

Optical density measurement at 600 nm was used for culture growth monitoring. Samples were processed by the procedure as published previously by Patakova et al. [17]. Substrate consumption and metabolite production were detected and quantified using HPLC with refractive index detection (Agilent Series 1200 HPLC, Agilent, Santa Clara, CA, USA). Sample preparation and analysis were performed identically to Patakova et al. [17].

### Microscopy, fluorescent staining, and flow cytometry

Cell morphology was determined in the native culture using phase contrast microscopy (BX51, Olympus, Tokio, Japan) using 400× and 1000× magnification. Cell culture viability and the amount of endospores were determined using flow cytometry (BD Accuri C6, Accuri Cytometers Inc., Ann Arbor, MI, USA) combined with PI (Sigma-Aldrich) and CFDA (Sigma-Aldrich) fluorescent staining using protocol published in Branska et al. [36].

### DNA extraction and sequencing

DNeasy UltraClean Microbial Kit (Qiagen, Hilden, Germany) was used for genomic DNA extraction. DNA was extracted from an exponentially growing culture; the quality of isolated genomic DNA was controlled using a nanodrop machine (DeNovix, Wilmington, DE, USA). Library construction and sequencing of the sample was performed by CEITEC Genomics core facility (Brno, Czechia) on Illumina NextSeq, pair-end, 150 bp.

### RNA extraction and sequencing

High Pure RNA Isolation Kit (Roche, Basel, Switzerland) was used for total RNA isolation from samples. The MICROB*Express*™ Bacterial mRNA Enrichment Kit (Ambion, Austin, TX, USA) was used for ribosomal RNAs’ depletion from total RNA samples. All RNA samples were stored at − 70 °C without next defrosting to prevent freeze–thaw damage. For control of quality of extracted total RNA, depleted mRNA, and to prevent DNA contaminations, an Agilent 2100 bioanalyzer with the RNA 6000 Nano Kit (Agilent, Santa Clara, CA, USA) in combination with routine spectrophotometric control on nanodrop machine (DeNovix, Wilmington, DE, USA) was used. Library construction and sequencing of samples were performed by CEITEC Genomics core facility (Brno, Czechia) on Illumina NextSeq, single-end, 75 bp.

### Bioinformatics analysis

The quality assessment of sequencing data (DNA and RNA) after all processing steps was done using FastQC in combination with MultiQC to summarize the reports across all samples [48]. Adapter and quality trimming was performed using Trimmomatic [49]. For the genome reassembly, reads from DNA sequencing were mapped to the previous genome sequence CP011966.2 with BWA [50]. The new assembly was constructed with Pilon [51]. Our improved assembly was used as a reference for the second mapping of reads and the second round of assembly polishing with Pilon. The resulting assembly was uploaded to GenBank as CP011966.3 version of the *C. beijerinckii* NRRL B-598 genome. RNA-Seq reads were cleansed of reads corresponding to 16S and 23S rRNA using SortMeRNA [52] and the SILVA database [53] of known bacterial 16S and 23S rRNA genes to simplify the following mapping task that was performed with STAR [54]. Resulting SAM (Sequence Read Alignment/Map) files were indexed and transformed into more compact BAM (Binary Read Alignment/Map) format using SAMtools [55].

The R/Bioconductor featureCounts function included in the Rsubread package [56] was used to compute count tables. Differential analysis was performed on raw count tables with the R/Bioconductor DESeq2 package [57] using DESeq2 built-in normalization. For the analysis of adjacent time-points presented in Venn diagrams, all samples were normalized at once. For separate analysis of particular time-points against the reference time-point, only compared samples were used for normalization. Visual comparison of samples was performed via t-SNE dimensionality reduction of a count table after regularized log transformation using the Rtsne [58] and ggplot2 [59] R packages. Venn diagrams and heatmaps representing transcription of selected genes using *Z* scores were generated with R packages VennDiagram [60] and gplots, respectively. Time series and bar plots were generated with Matlab 2017b and gplots.

The GO annotation map file was compiled from annotations obtained with QuickGO [61] and Blast2GO [62] with custom-made R/Bioconductor scripts using functions from the genomeIntervals, Biostrings, and topGO packages [29]. Basic statistics of the GO annotation were computed using the dnet and igraph R packages [63, 64]. GO enrichment analysis was performed using the topGO package [29].

## Supplementary information

**Additional file 1.** Comparison of cultivation and fermentation characteristics of *Clostridium beijerinckii* NRRL B-598 during standard cultivation and butanol shock.

**Additional file 2.** *Clostridium beijerinckii* NRRL B-598 microphotograph.

**Additional file 3.** Differences between assemblies.

**Additional file 4.** Differences between genome annotations.

**Additional file 5.** *Clostridium beijerinckii* NRRL B-598 Gene Ontology annotation

**Additional file 6.** A brief overview of the *C. beijerinckii* NRRL B-598 GO annotation.

**Additional file 7.** Quality of RNA-Seq reads and mapping.

**Additional file 8.** Differential expression analysis of adjacent time-points using MA plots.

**Additional file 9.** Complete differential expression analysis of adjacent time-points.

**Additional file 10.** Genes under enriched GO terms in cluster 1.

**Additional file 11.** Genes under enriched GO terms in cluster 2.

**Additional file 12.** Genes under enriched GO terms in cluster 3.

## Data Availability

The genome assembly referred in this paper is the version CP011966.3. The genome sequencing and RNA-Seq data have been deposited in the NCBI Sequence Read Archive (SRA) under the accession number SRP033480 (SRX6419026 for F replicates, SRX6419027 for G replicates, and SRX6419139 for genome resequencing, respectively).

## Abbreviations

ABE: acetone–butanol–ethanol
BP: biological process
CC: cellular component
CFDA: carboxyfluorescein diacetate
COG: clusters of orthologous genes
FC: flow cytometry
GO: gene ontology
HPLC: high-pressure liquid chromatography
MF: molecular function
OD: optical density
PI: propidium iodide
R-M: restriction-modification
SASPs: small, acid-soluble proteins
SNV: single-nucleotide variant
