# Supplementary material for: A transcriptional response of Clostridium beijerinckii NRRL B-598 to a butanol shock
Source: Biotechnol Biofuels. 2019 Oct 13;12:243. doi: 10.1186/s13068-019-1584-7 (PMC6790243; doi:10.1186/s13068-019-1584-7)

Additional file 1: Comparison of cultivation and fermentation characteristics of *Clostridium beijerinckii* NRRL B-598 during standard cultivation and butanol shock

The concentration of glucose, solvents and acids during ABE fermentation using HPLC. Solid line represent fermentation during the butanol shock experiment. Dashed line represent fermentation during a standard cultivation from our previous study (Sedlar et al., 2018).

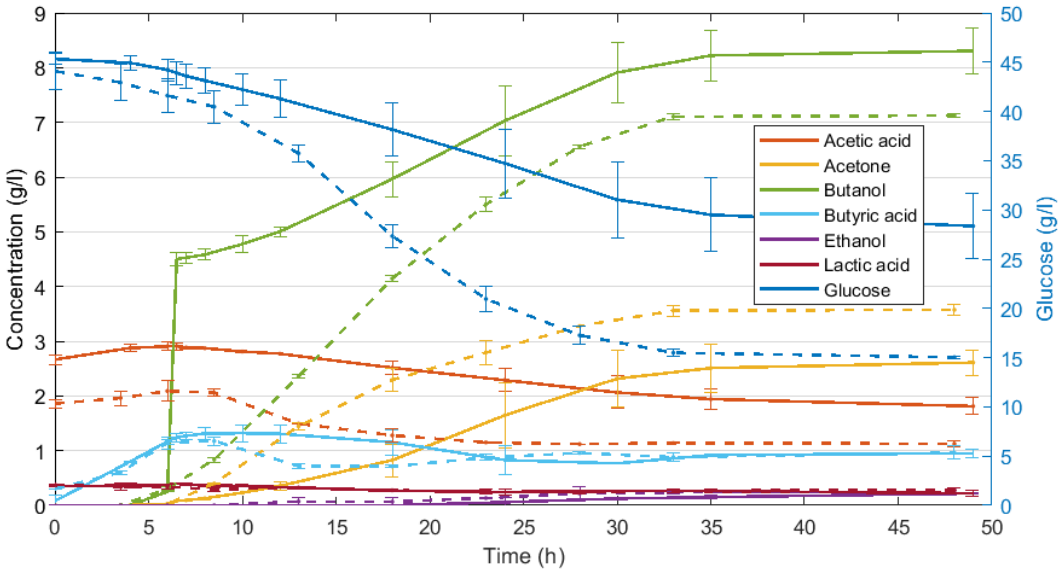

Flow cytometry – the distribution of cells within the population according to their fluorescence pattern for combined staining using PI and CFDA. Replicates B, C represent data from a standard cultivation from our previous study (Sedlar et al., 2018). Replicates F, G represent data from the butanol shock study.

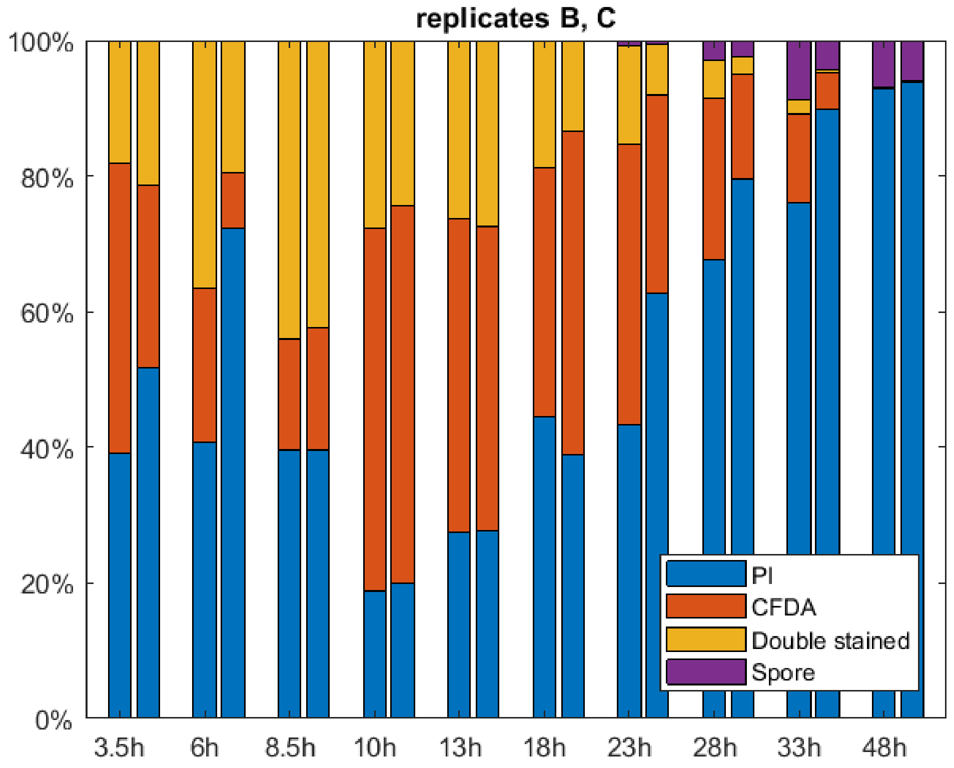

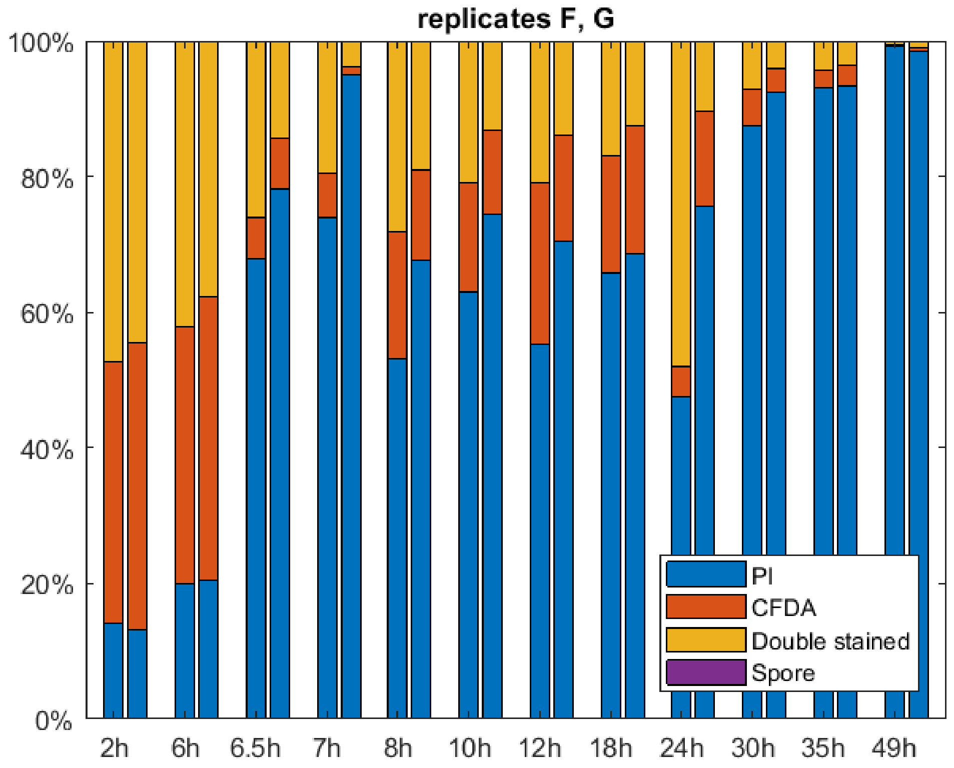

pH curve for the butanol shock experiment (solid line) and for a standard cultivation from our previous study (Sedlar et al., 2018) (dashed line).

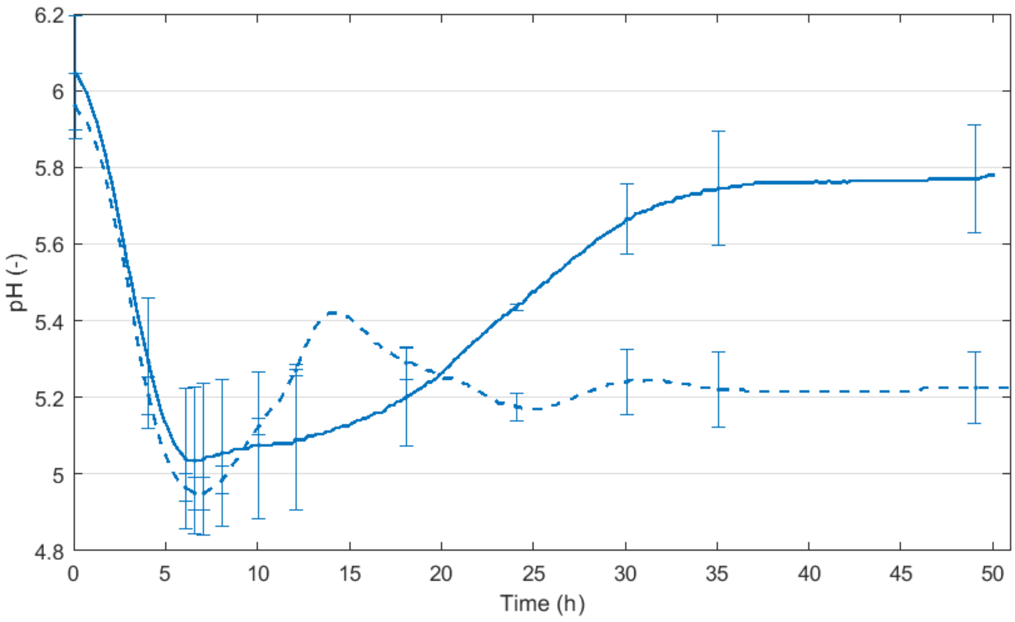

Cell growth measured as optical density at 600 nm for the butanol shock cultivation (solid line) and for a standard cultivation from our previous study (Sedlar et al., 2018) (dashed line).

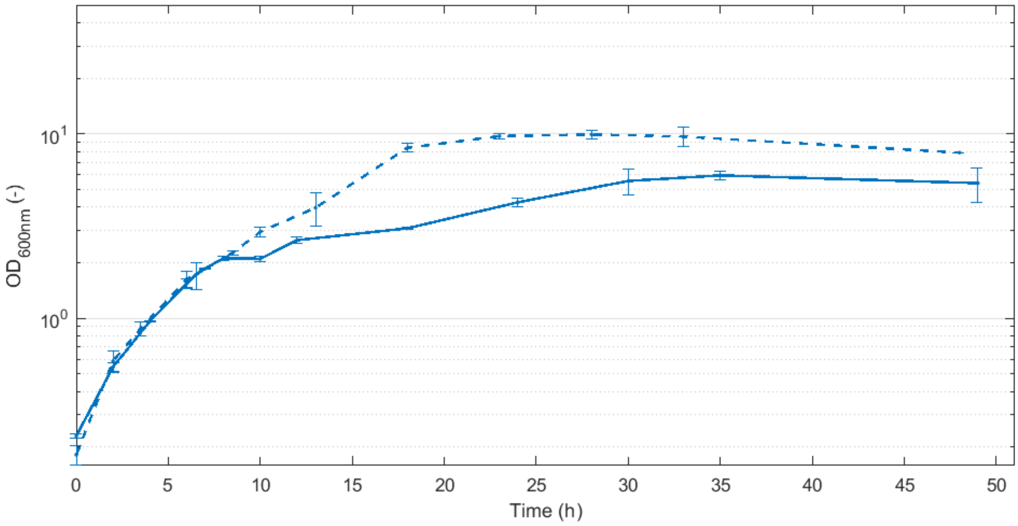

Supplement: Supplementary file 1 — Additional file 1. Comparison of cultivation and fermentation characteristics of Clostridium beijerinckii NRRL B-598 during standard cultivation and butanol shock. [file 13068_2019_1584_MOESM1_ESM.pdf]
