# Supplementary material for: A transcriptional response of Clostridium beijerinckii NRRL B-598 to a butanol shock
Source: Biotechnol Biofuels. 2019 Oct 13;12:243. doi: 10.1186/s13068-019-1584-7 (PMC6790243; doi:10.1186/s13068-019-1584-7)

Additional file 2: *Clostridium beijerinckii* NRRL B-598 microphotograph

Clostridium beijerinckii NRRL B-598 microphotograph at the 24th hour of cultivation (18 h after addition of butanol).

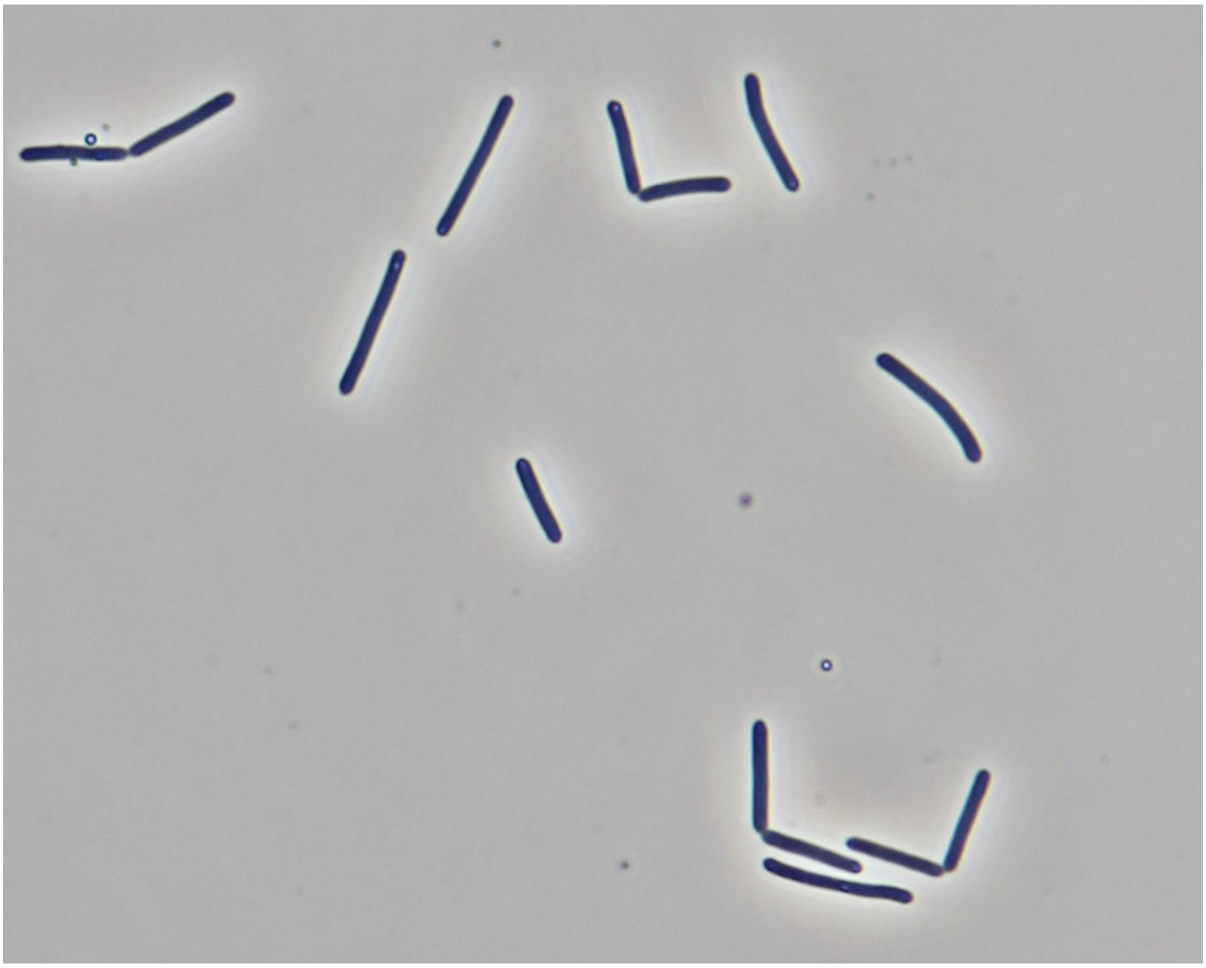

Supplement: Supplementary file 2 — Additional file 2. Clostridium beijerinckii NRRL B-598 microphotograph. [file 13068_2019_1584_MOESM2_ESM.pdf]
