# Supplementary material for: A transcriptional response of Clostridium beijerinckii NRRL B-598 to a butanol shock
Source: Biotechnol Biofuels. 2019 Oct 13;12:243. doi: 10.1186/s13068-019-1584-7 (PMC6790243; doi:10.1186/s13068-019-1584-7)

## Additional file 6: A brief overview of the *C. beijerinckii* NRRL B-598 GO annotation

**Manually curated annotation:** Levels (longest distance from the root) of 18,020 unique GO terms assignments in C. beijerinckii NRRL B-598 GO annotation divided into GO categories: BP = Biological Process, CC = Cellular Component, and MF = molecular function.

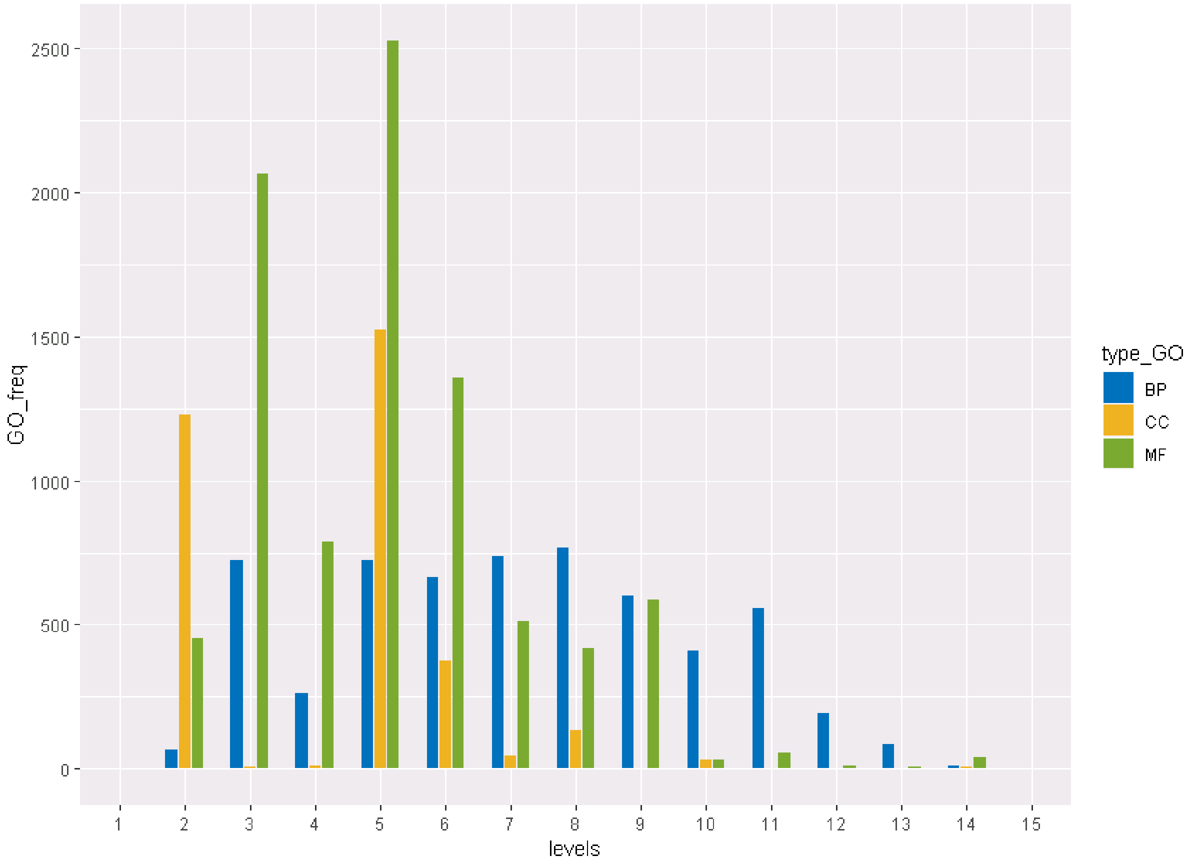

**Manually curated annotation:** Levels (longest distance from the root) of 1,702 distinct GO terms associated with C. beijerinckii NRRL B-598 genome divided into GO categories: BP = Biological Process, CC = Cellular Component, and MF = molecular function.

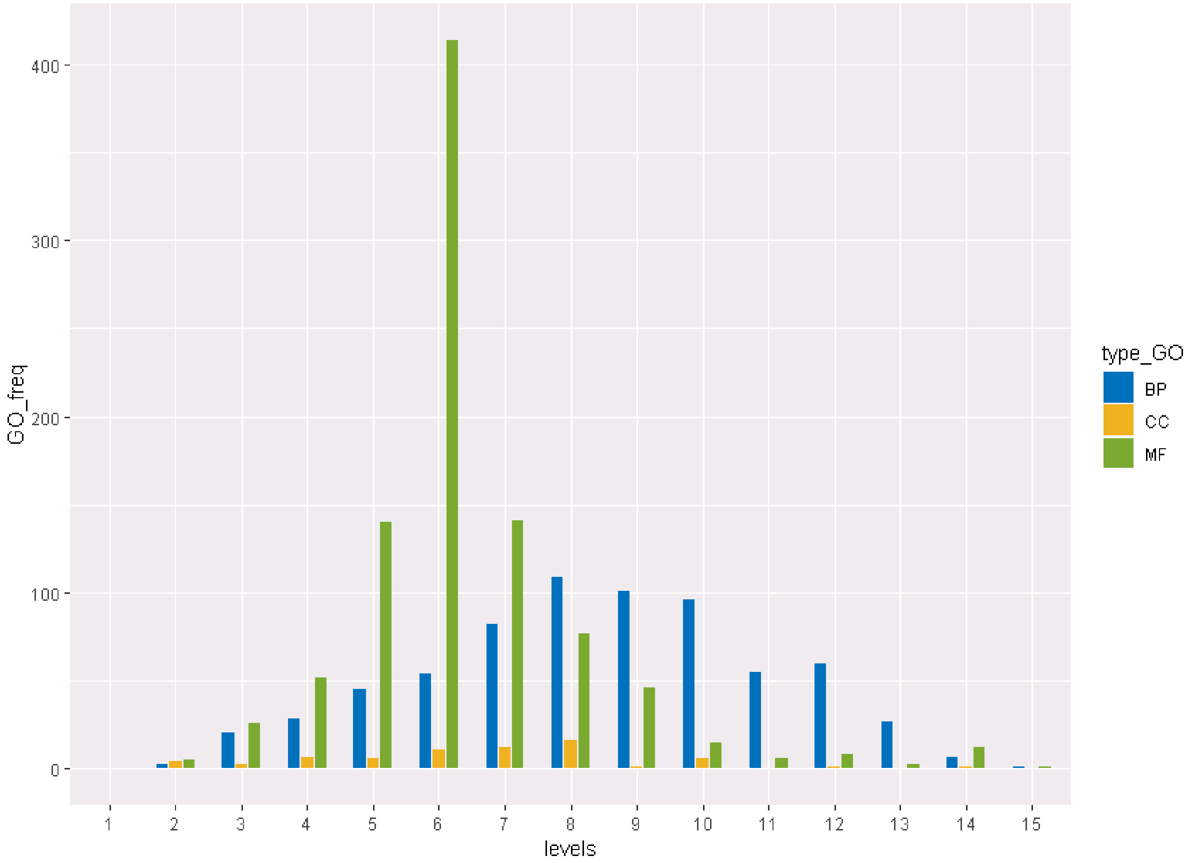

**Default database search annotation:** Levels (longest distance from the root) of 16,271 unique GO terms assignments in C. beijerinckii NRRL B-598 GO annotation divided into GO categories: BP = Biological Process, CC = Cellular Component, and MF = molecular function.

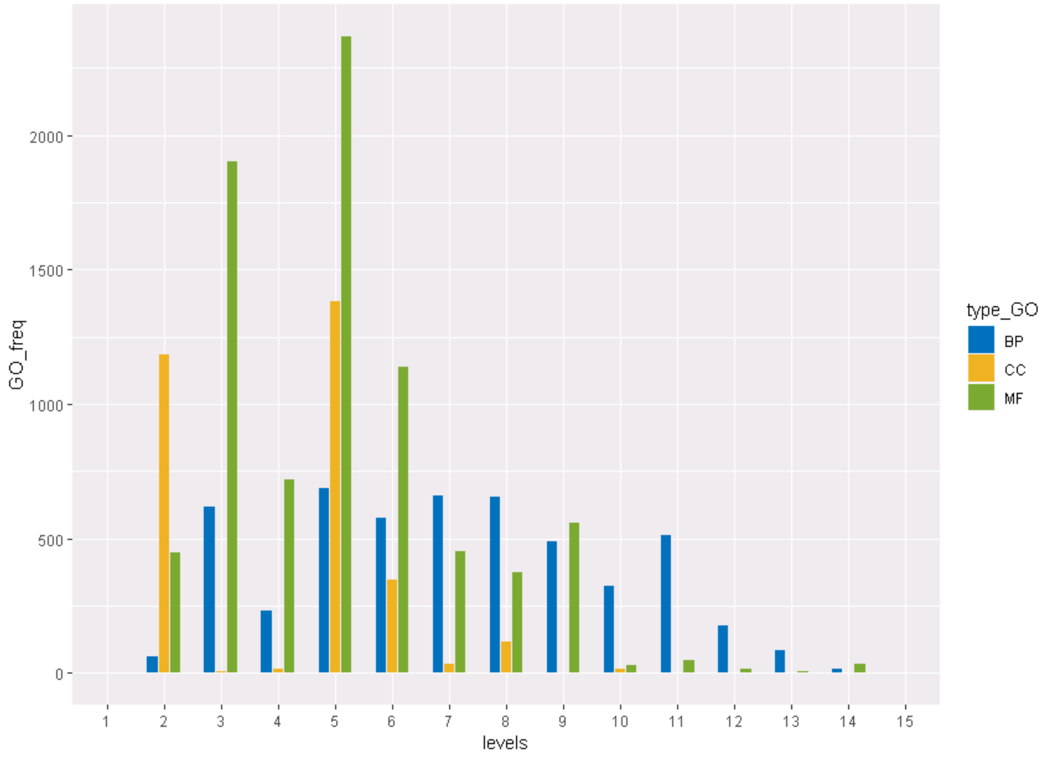


**Default database search annotation:** Levels (longest distance from the root) of 1,507 distinct GO terms associated with C. beijerinckii NRRL B-598 genome divided into GO categories: BP = Biological Process, CC = Cellular Component, and MF = molecular function.

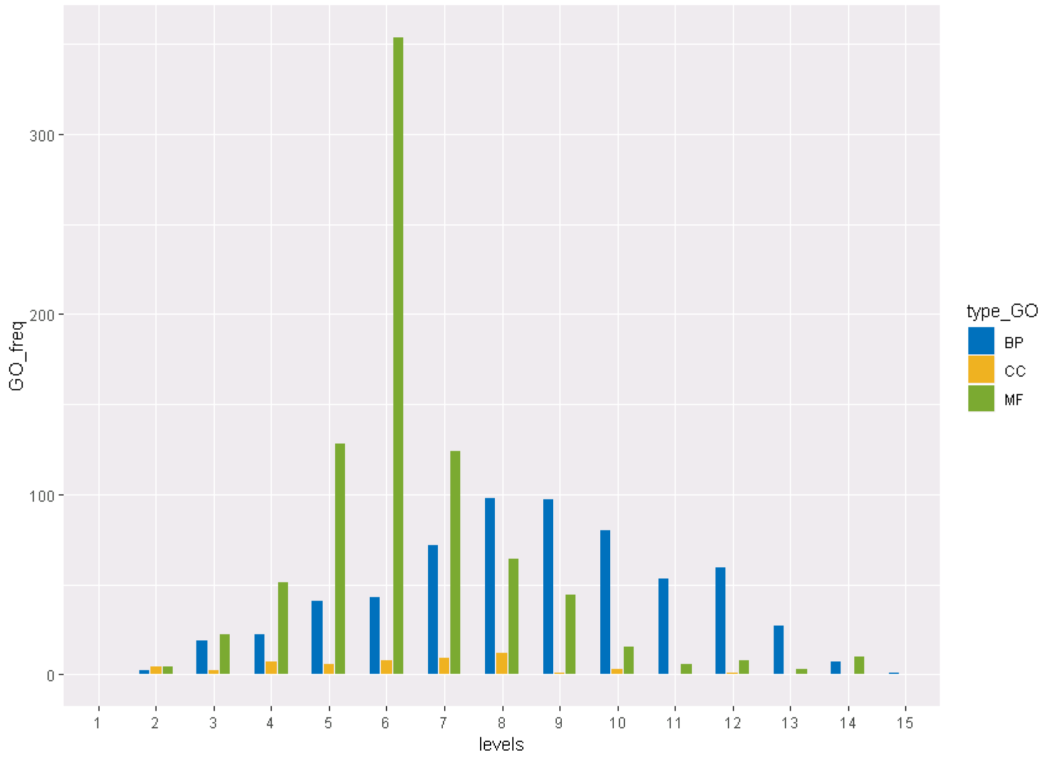

Supplement: Supplementary file 6 — Additional file 6. A brief overview of the C. beijerinckii NRRL B-598 GO annotation. [file 13068_2019_1584_MOESM6_ESM.pdf]
