# Supplementary material for: A transcriptional response of Clostridium beijerinckii NRRL B-598 to a butanol shock
Source: Biotechnol Biofuels. 2019 Oct 13;12:243. doi: 10.1186/s13068-019-1584-7 (PMC6790243; doi:10.1186/s13068-019-1584-7)

Additional file 7: Quality of RNA-Seq reads and mapping

(a) The total number of reads in particular samples. The color of stacked bars distinguishes between non-rRNA and rRNA reads. (b) Mapping statistics of reads – percentages of uniquely mapped, multi-mapped, and unmapped non-rRNA reads.

a

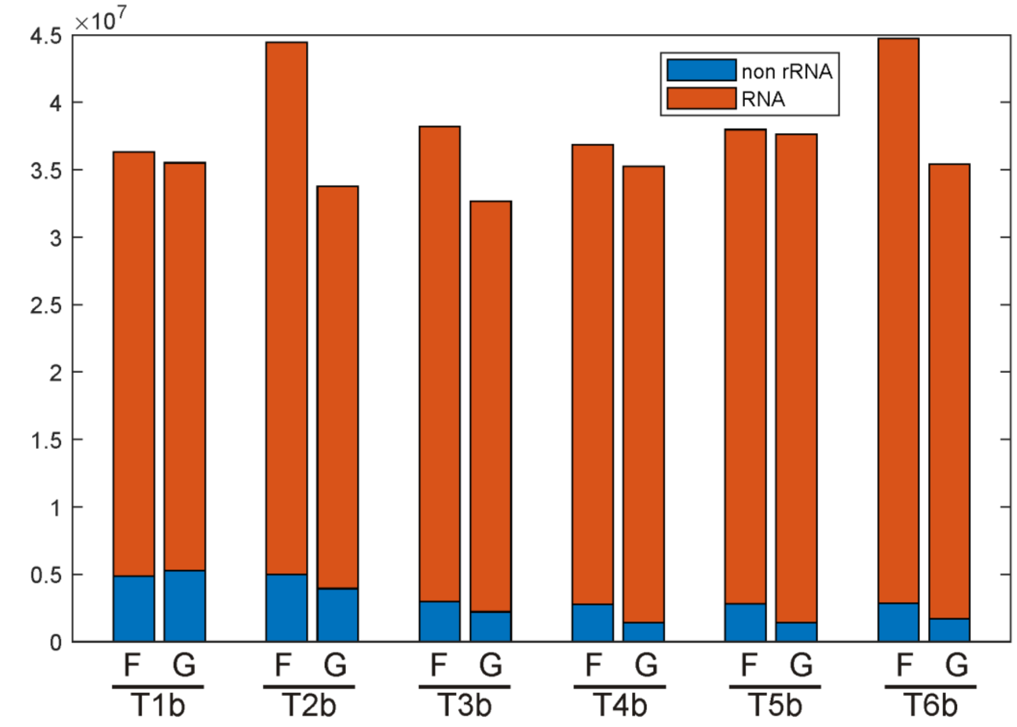

b

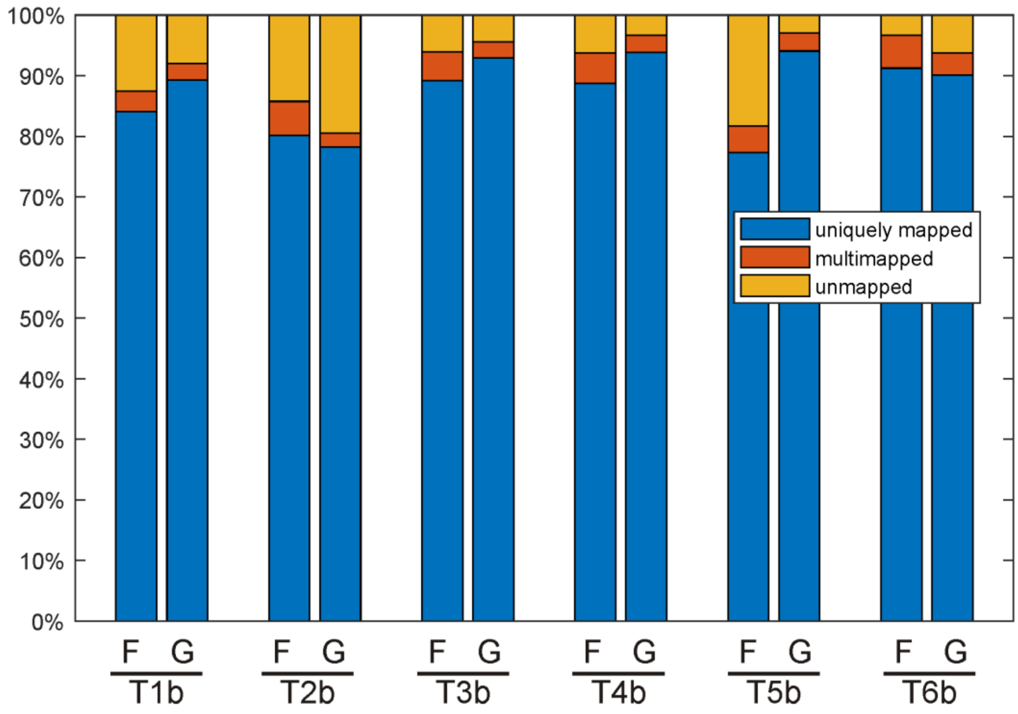

Supplement: Supplementary file 7 — Additional file 7. Quality of RNA-Seq reads and mapping. [file 13068_2019_1584_MOESM7_ESM.pdf]
