# Supplementary material for: A transcriptional response of Clostridium beijerinckii NRRL B-598 to a butanol shock
Source: Biotechnol Biofuels. 2019 Oct 13;12:243. doi: 10.1186/s13068-019-1584-7 (PMC6790243; doi:10.1186/s13068-019-1584-7)

Additional file 8: Differential analysis of adjacent time-points using MA plots

MA plots showing statistically differentially expressed genes in color (padj < 0.05). Color coding respect the color coding used in Venn diagrams in Figure 3.

$T_b0 \rightarrow T_b1$

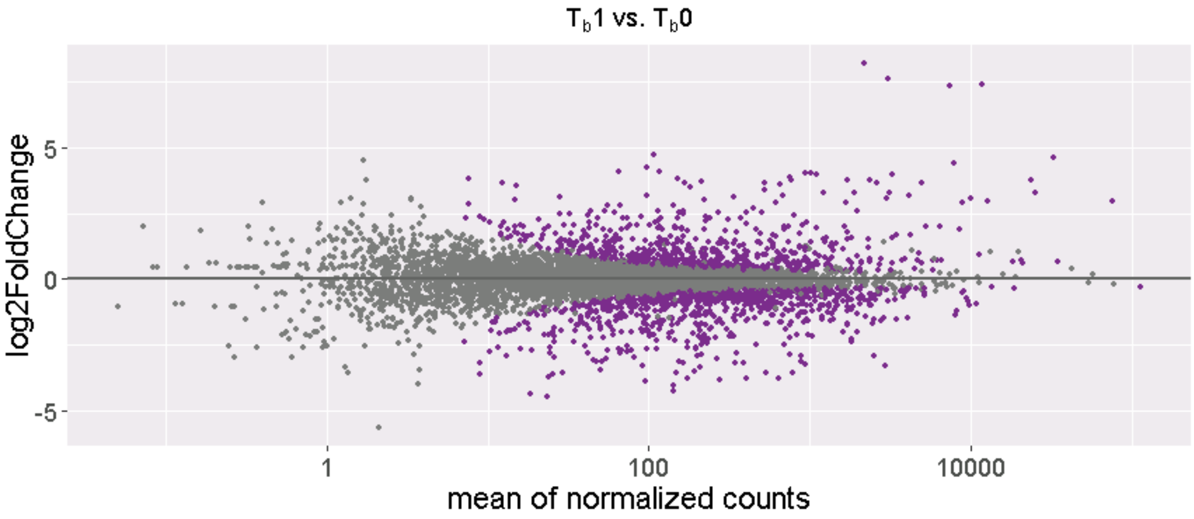

$T_b1 \rightarrow T_b2$

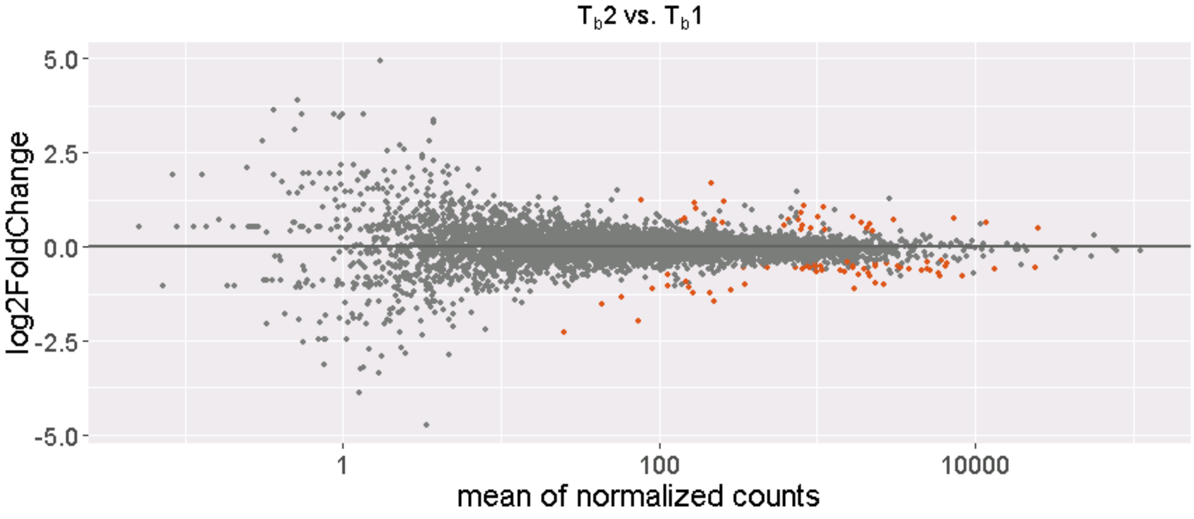

$T_b2 \rightarrow T_b3$

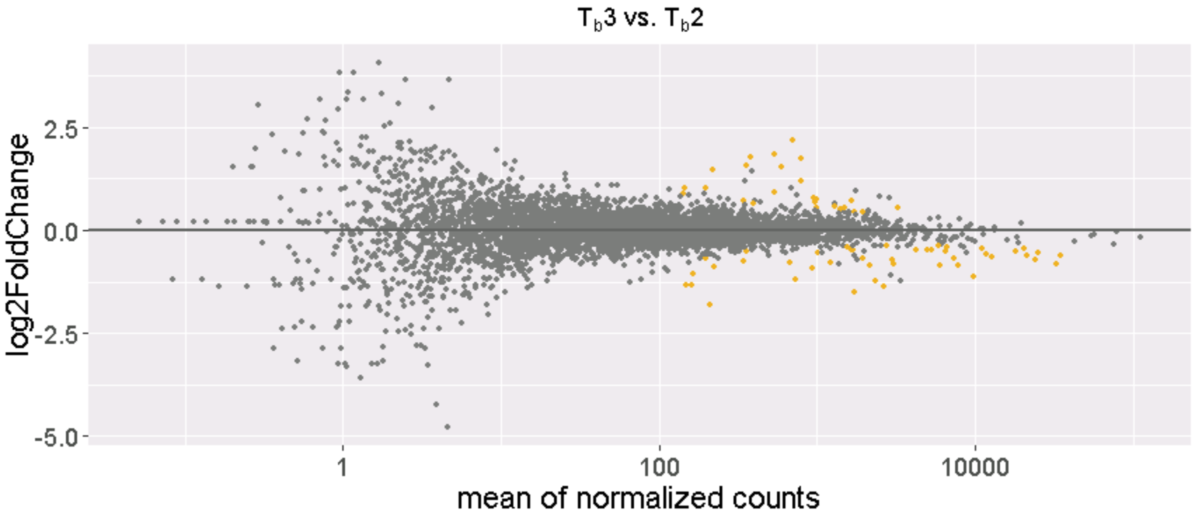

$T_b3 \rightarrow T_b4$

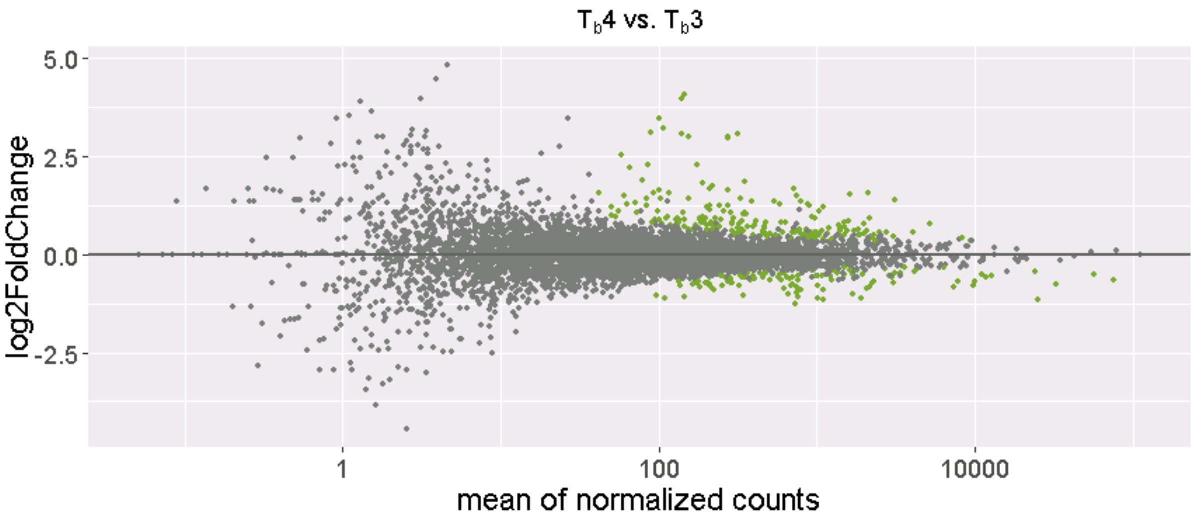

$T_b4 \rightarrow T_b5$

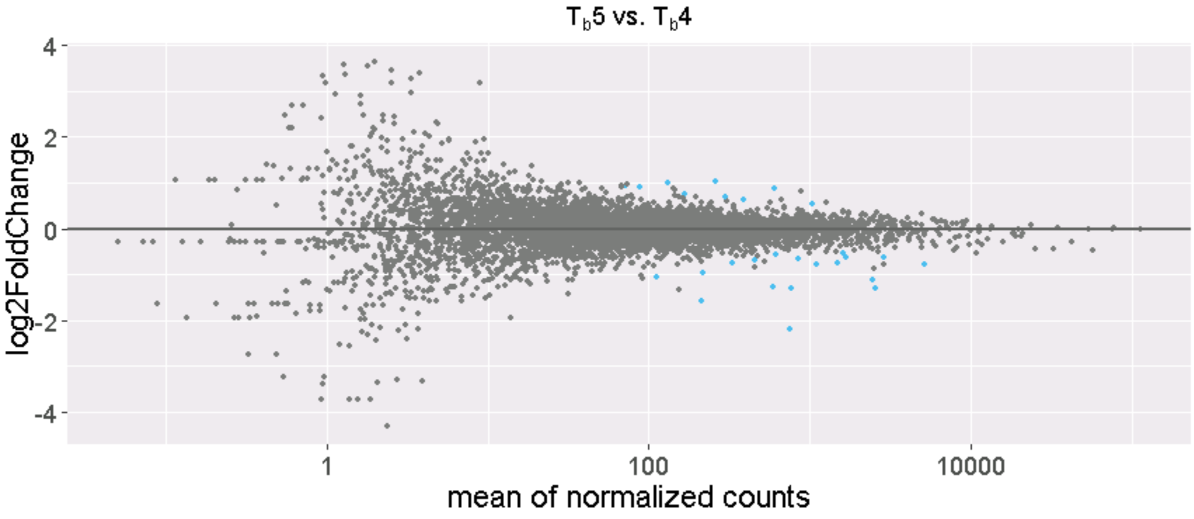

Supplement: Supplementary file 8 — Additional file 8. Differential expression analysis of adjacent time-points using MA plots. [file 13068_2019_1584_MOESM8_ESM.pdf]
